# Multiple pathways impact the swarming motility of *Pseudomonas fluorescens* Pf0-1

**DOI:** 10.1128/spectrum.00166-24

**Published:** 2024-04-30

**Authors:** Alexander B. Pastora, Kara M. Rzasa, George A. O’Toole

**Affiliations:** 1 Department of Microbiology and Immunology, Geisel School of Medicine at Dartmouth, Hanover, New Hampshire, USA; 2 Thayer School of Engineering at Dartmouth, Hanover, New Hampshire, USA; Emory University School of Medicine, Atlanta, Georgia, USA

**Keywords:** swarming, regulation, *Pseudomonas fluorescens*, biosurfactant, flagellum

## Abstract

Swarming motility in pseudomonads typically requires both a functional flagellum and the production/secretion of a biosurfactant. Published work has shown that the wild-type *Pseudomonas fluorescens* Pf0-1 is swarming deficient due to a point mutation in the *gacA* gene, which until recently was thought to inactivate rather than attenuate the Gac/Rsm pathway. As a result, little is known about the underlying mechanisms that regulate swarming motility by *P. fluorescens* Pf0-1. Here, we demonstrate that a Δ*rsmA* Δ*rsmE* Δ*rsmI* mutant, which phenotypically mimics Gac/Rsm pathway overstimulation, is proficient at swarming motility. RsmA and RsmE appear to play a key role in this regulation. Transposon mutagenesis of the Δ*rsmA* Δ*rsmE* Δ*rsmI* mutant identified multiple factors that impact swarming motility, including pathways involved in flagellar synthesis and biosurfactant production/secretion. We find that loss of genes linked to biosurfactant Gacamide A biosynthesis or secretion impacts swarming motility, as does loss of the alternative sigma factor FliA, which results in a defect in flagellar function. Collectively, these findings provide evidence that *P. fluorescens* Pf0-1 can swarm if the Gac/Rsm pathway is activated, highlight the regulatory complexity of swarming motility in this strain, and demonstrate that the cyclic lipopeptide Gacamide A is utilized as a biosurfactant for swarming motility.

**IMPORTANCE:**

Swarming motility is a coordinated process that allows communities of bacteria to collectively move across a surface. For *P. fluorescens* Pf0-1, this phenotype is notably absent in the parental strain, and to date, little is known about the regulation of swarming in this strain. Here, we identify RsmA and RsmE as key repressors of swarming motility *via* modulating the levels of biosurfactant production/secretion. Using transposon mutagenesis and subsequent genetic analyses, we further identify potential regulatory mechanisms of swarming motility and link Gacamide A biosynthesis and transport machinery to swarming motility.

## INTRODUCTION

Swarming motility is a highly coordinated form of movement, generally requiring a functional flagellum and a wetting agent, that allows for the rapid translocation of surface-associated bacterial communities. Swarming has been characterized in a variety of Gram-positive and Gram-negative bacteria including *Bacillus subtilis*, *Escherichia coli*, *Pseudomonas aeruginosa*, *Salmonella enterica*, and *Serratia marcescens* (1–15). Regulation of swarming motility has been extensively studied in the pseudomonads (16–28).

For *Pseudomonas fluorescens*, swarming motility is important for efficient colonization of plant roots and rapid movement toward root exudates (29–32). Interestingly, the wild-type *P. fluorescens* Pf0-1 strain, which was isolated from nutrient-rich sandy-loam soil (33), is deficient for swarming motility due to a N109P point mutation in the *gacA* gene, which was previously thought to completely abolish the function of the Gac/Rsm pathway (34, 35). However, a recent study determined that the *P. fluorescens* Pf0-1 Gac/Rsm pathway could be stimulated by the overproduction of the upstream histidine kinase GacS, suggesting that the N109P mutation in the *gacA* gene attenuates rather than abolishes the Gac/Rsm pathway of *P. fluorescens* Pf0-1 and that overstimulation of the pathway could be achieved via chromosomal deletions of *rsmA*, *rsmE*, and *rsmI* genes (36). The loss of these *rsm* genes results in overstimulation because the respective Rsm proteins are not present to bind and regulate the translation of their target mRNAs (37, 38).

Here we show that the Δ*rsmA* Δ*rsmE* Δ*rsmI* triple mutant of *P. fluorescens* Pf0-1 is capable of swarming motility. We demonstrate that loss of RsmA and RsmE functions is sufficient to induce swarming by *P. fluorescens* Pf0-1. Starting with the Δ*rsmA* Δ*rsmE* Δ*rsmI* mutant, we use a genetic screen and subsequent molecular genetic studies to identify pathways involved in swarming motility. These pathways impact flagellar motility and link the biosynthesis of the biosurfactant Gacamide A and its secretion machinery with swarming motility.

## RESULTS

### The Gac/Rsm pathway regulates swarming motility in a RsmA- and RsmE-dependent manner

To assess the role of the Gac/Rsm pathway for swarming motility, we utilized a strain deficient in the small proteins RsmA, RsmE, and RsmI (Rsm proteins) in *P. fluorescens* Pf0-1, which was previously demonstrated to mimic overstimulation of the Gac/Rsm Pathway (36). We probed this strain for flagellar function using a Tris-buffered minimal medium supplemented with L-arginine as the sole carbon source (KA medium) supplemented with 0.3% agar (designated “swim agar” for assessing swimming motility) or 0.5% agar (designated “swarm agar” for assessing swarming motility).

As previously shown, low percentage agar media, including swarm agar, can also be used to probe for biosurfactant production/secretion by pseudomonads. Biosurfactant production is phenotypically defined as a translucent liquid zone radiating from the inoculum that then forms the leading edge of the subsequent swarm zone (11, 39, 40). In this study, we designate this phenotype on swarm agar as the “biosurfactant zone.” As a negative control, we included a strain deficient in FleQ function (a Δ*fleQ* mutant), which has previously been shown to lack flagellar motility in multiple pseudomonads (24, 27, 41–44).

**Fig 1:**
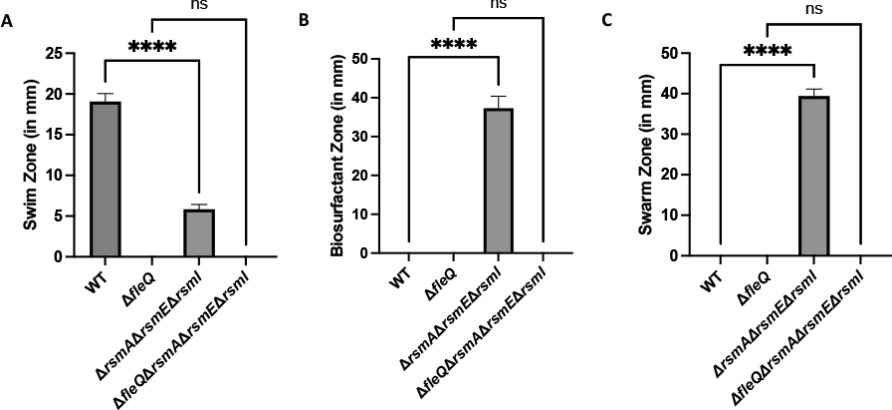
A strain lacking the Rsm proteins swarms. (**A**) Swim zone (in millimeters) of the WT strain, Δ*fleQ* single mutant, Δ*rsmA* Δ*rsmE* Δ*rsmI* triple mutant, and a Δ*fleQ* Δ*rsmA* Δ*rsmE* Δ*rsmI* quadruple mutant after toothpick inoculation on KA minimal medium supplemented with 0.3% agar followed by 24-h growth at 30°C. (**B**) The biosurfactant zone (in millimeters) of the WT strain, Δ*fleQ* single mutant, Δ*rsmA* Δ*rsmE* Δ*rsmI* triple mutant, and a Δ*fleQ* Δ*rsmA* Δ*rsmE* Δ*rsmI* quadruple mutant after inoculation of 2.5 µL of overnight culture on the surface of KA minimal medium supplemented with 0.5% agar followed by 24-h growth at 30°C. (**C**) Swarm zone (in millimeters) of the WT strain, Δ*fleQ* single mutant, Δ*rsmA* Δ*rsmE* Δ*rsmI* triple mutant, and a Δ*fleQ* Δ*rsmA* Δ*rsmE* Δ*rsmI* quadruple mutant after inoculation of 2.5 µL of overnight culture on the surface of KA minimal medium supplemented with 0.5% agar followed by 24-h growth at 30°C then 24-h growth at room temperature. Statistical significance for this figure was determined using one-way ANOVA with Tukey’s multiple comparisons tests. ****P < 0.0001. All error bars represent standard deviation.

After 24-h growth on swim agar at 30°C, the Δ*rsmA* Δ*rsmE* Δ*rsmI* triple mutant, which was capable of swimming, showed significantly less swimming motility compared to the wild-type (WT) strain (Fig. 1A). This reduced swimming phenotype was reported previously by Pastora and O’Toole (36) and attributed to differences in planktonic growth (36). By contrast, the Δ*fleQ* and Δ*fleQ* Δ*rsmA* Δ*rsmE* Δ*rsmI* mutant strains were completely deficient for swimming motility (Fig. 1A).

The Δ*rsmA* Δ*rsmE* Δ*rsmI* mutant produced a biosurfactant zone while the WT strain and FleQ-deficient mutants did not produce a measurable biosurfactant zone after 24-h growth on swarm agar at 30°C (Fig. 1B). Interestingly, the Δ*rsmA* Δ*rsmE* Δ*rsmI* mutant also produced a sizeable but smaller biosurfactant zone when grown for 24 h at room temperature (Fig. S1A). Since the initial 24-h incubation time was not sufficient to induce swarming motility in any of the tested strains or temperature conditions, we subsequently incubated the strains for an additional 24 h at room temperature. After 24-h growth on swarm agar at 30°C and then 24-h growth at room temperature, the Δ*rsmA* Δ*rsmE* Δ*rsmI* mutant displayed motility on swarm agar, whereas the other strains were deficient for swarming motility (Fig. 1C). The Δ*rsmA* Δ*rsmE* Δ*rsmI* mutant produced a similar swarm zone when grown on swarm agar for 48 h at room temperature but produced a significantly smaller swarm zone when grown on swarm agar for 48 h at 30°C (Fig. S1B). Interestingly, the Δ*rsmA* Δ*rsmE* Δ*rsmI* mutant swims, produces biosurfactant, and eventually swarms on swim agar (Fig. S2), although to a lesser extent than on swarm agar.

**Fig 2:**
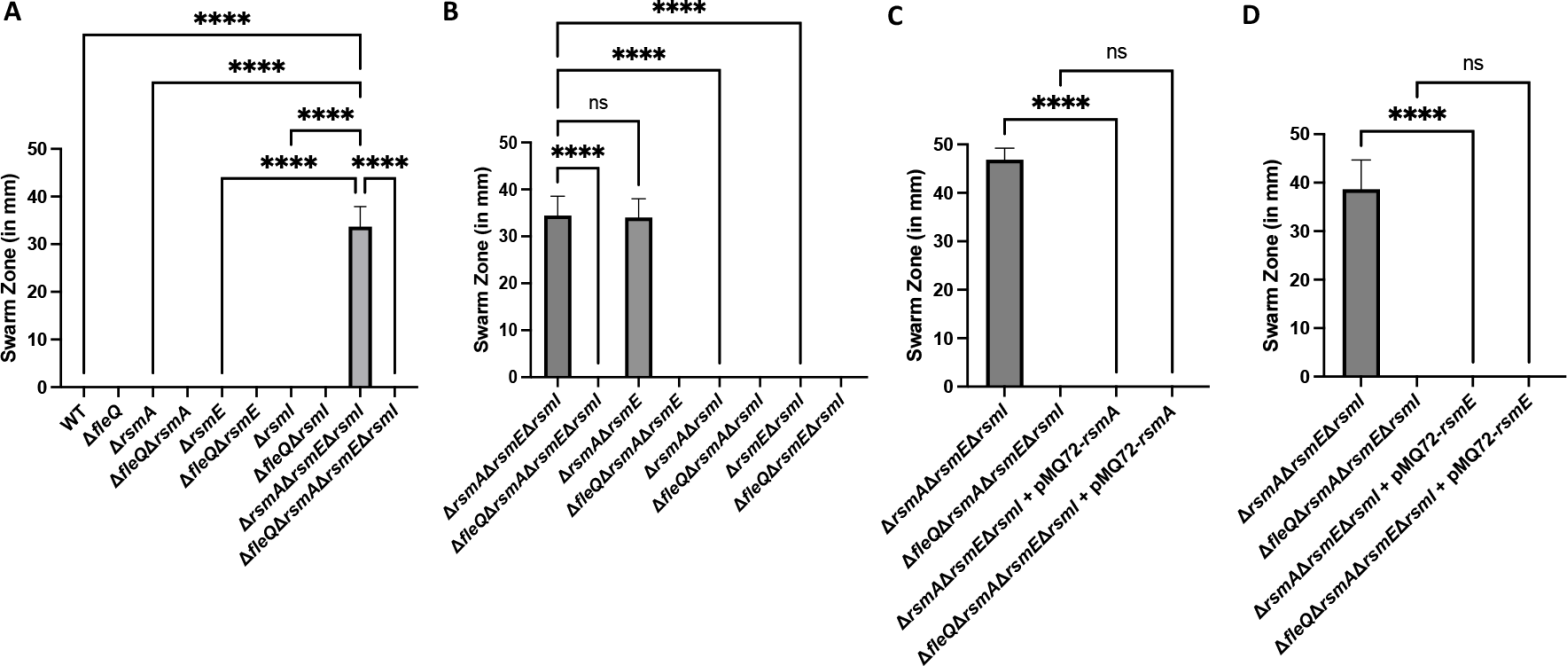
A strain deficient for both RsmA and RsmE shows swarming motility. (**A**) Swarm zone (in millimeters) of the WT strain, Δ*fleQ* single mutant, Δ*rsmA* single mutant, Δ*fleQ* Δ*rsmA* double mutant, Δ*rsmE* single mutant, Δ*fleQ* Δ*rsmE* double mutant, Δ*rsmI* single mutant, Δ*fleQ* Δ*rsmI* double mutant, Δ*rsmA* Δ*rsmE* Δ*rsmI* triple mutant, and a Δ*fleQ* Δ*rsmA* Δ*rsmE* Δ*rsmI* quadruple mutant after inoculation of 2.5 µL of overnight culture on the surface of KA minimal medium supplemented with 0.5% agar followed by 24-h growth at 30°C then 24-h growth at room temperature. (**B**) Swarm zone (in millimeters) of the Δ*rsmA* Δ*rsmE* Δ*rsmI* triple mutant, Δ*fleQ* Δ*rsmA* Δ*rsmE* Δ*rsmI* quadruple mutant, Δ*rsmA* Δ*rsmE* double mutant, Δ*fleQ* Δ*rsmA* Δ*rsmE* triple mutant, Δ*rsmA* Δ*rsmI* double mutant, Δ*fleQ* Δ*rsmA* Δ*rsmI* triple mutant, Δ*rsmE* Δ*rsmI* double mutant, and Δ*fleQ* Δ*rsmE* Δ*rsmI* triple mutant after inoculation of 2.5 µL of overnight culture on the surface of KA minimal medium supplemented with 0.5% agar followed by 24-h growth at 30°C and then 24-h growth at room temperature. (**C**) Swarm zone (in millimeters) of the Δ*rsmA* Δ*rsmE* Δ*rsmI* mutant, Δ*fleQ* Δ*rsmA* Δ*rsmE* Δ*rsmI* mutant, Δ*rsmA* Δ*rsmE* Δ*rsmI* mutant + pMQ72 *rsmA*, and Δ*fleQ* Δ*rsmA* Δ*rsmE* Δ*rsmI* mutant + pMQ72 *rsmA* after inoculation of 2.5 µL of overnight culture on the surface of KA minimal medium supplemented with 0.5% agar followed by 24-h growth at 30°C and then 24-h growth at room temperature. (**D**) The swarm zone (in millimeters) of the Δ*rsmA* Δ*rsmE* Δ*rsmI* mutant, Δ*fleQ* Δ*rsmA* Δ*rsmE* Δ*rsmI* mutant, Δ*rsmA* Δ*rsmE* Δ*rsmI* mutant + pMQ72 *rsmE*, and Δ*fleQ* Δ*rsmA* Δ*rsmE* Δ*rsmI* mutant + pMQ72 *rsmE* after inoculation of 2.5 µL of overnight culture on the surface of KA minimal medium supplemented with 0.5% agar followed by 24-h growth at 30°C and then 24-h growth at room temperature. Statistical significance for this figure was determined using one-way ANOVA with Tukey’s multiple comparisons tests. ****P < 0.0001. All error bars represent standard deviation.

To identify the Rsm proteins associated with swarming motility, we made chromosomal deletions of the individual genes coding for the Rsm proteins in the WT and FleQ-deficient background strains and assessed these strains on swarm agar. Loss of any individual Rsm protein was not sufficient to induce swarming motility (Fig. 2A). Analysis of these strains for swimming motility and biosurfactant production revealed that while the single *rsm* deletions in the WT background were proficient for swimming motility (Fig. S3A), none of these strains were able to produce a biosurfactant zone (Fig. S3B).

We made combinatorial chromosomal deletions of the genes coding for the various Rsm proteins in the WT and FleQ-deficient backgrounds and assessed these mutants for their ability to swarm. After growth for 24 h at 30°C and then 24 h at room temperature, the Δ*rsmA* Δ*rsmE* double mutant was able to swarm to levels similar to the Δ*rsmA* Δ*rsmE* Δ*rsmI* triple mutant, while the other combinatorial deletions were deficient for swarming motility (Fig. 2B). Analysis of these strains for swimming motility and biosurfactant production revealed that all deletions in the WT background were proficient for swimming motility (Fig. S4A), but of these strains, only the Δ*rsmA* Δ*rsmE* double and Δ*rsmA* Δ*rsmE* Δ*rsmI* triple mutants were proficient for biosurfactant production (Fig. S4B).

To further demonstrate the role of RsmA and RsmE in regulating swarming motility, we inserted the coding sequence of RsmA or RsmE into the arabinose-inducible shuttle vector pMQ72 directly downstream of the P*_BAD_* promoter (see Materials and Methods), transformed the constructs into the Δ*rsmA* Δ*rsmE* Δ*rsmI* and Δ*fleQ*Δ*rsmA* Δ*rsmE* Δ*rsmI* backgrounds and assessed these strains for their ability to swarm. After growth for 24 h at 30°C and then 24 h at room temperature on swarm agar without inducing conditions (no addition of arabinose), the Δ*rsmA* Δ*rsmE* Δ*rsmI* + pMQ72 *rsmA* and Δ*rsmA* Δ*rsmE* Δ*rsmI* + pMQ72 *rsmE* strains are completely deficient for swarming motility (Fig. 2C and D, respectively). Analysis of these strains revealed that Δ*rsmA* Δ*rsmE* Δ*rsmI* + pMQ72 *rsmA* and Δ*rsmA* Δ*rsmE* Δ*rsmI* + pMQ72 *rsmE* strains were proficient in swimming motility (Fig. S5A and B), and these strains had a statistically significant decrease in biosurfactant secretion compared to their parental strains (Fig. S5C and D).

### Transposon mutagenesis reveals genes required for swarming motility

Given our observation that the Δ*rsmA* Δ*rsmE* Δ*rsmI* strain is proficient for swarming motility, we generated a Tn*M* mariner transposon mutant library as previously described in Pastora and O’Toole (36). We initially probed this library for candidates that were able to swarm after 24 h of growth at 30°C on swarm agar (hyper-swarm candidates) or were completely deficient in swarming motility after 24 h of growth at 30°C and then 24 h of growth at room temperature on swarm agar (swarm-deficient candidates) and identified the approximate transposon insertion site and P*_TAC_* promoter orientation using arbitrary primed PCR.

**Fig 3:**
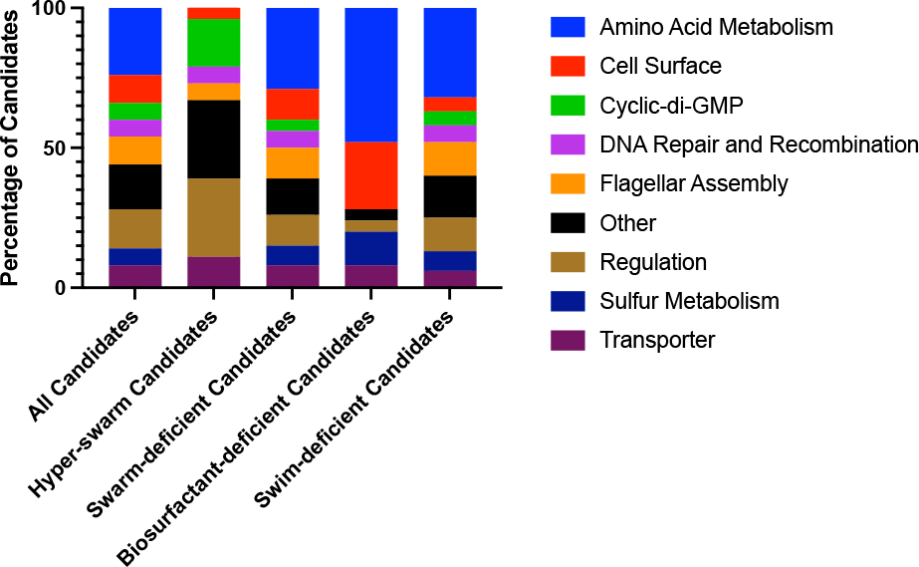
Summary of genetic loci identified by transposon mutagenesis. Stacked bar charts categorizing the candidates (*n* = 108) identified from the transposon mutagenesis. Candidates were phenotypically sub-grouped based on swarming motility into hyper-swarming (*n* = 18) or swarm deficient (*n* = 90). The swarm-deficient group was then sub-grouped based on a loss of biosurfactant production (*n* = 25) or a defect in swimming motility (*n* = 82), with a subset of candidates present in both groups.

These candidates were re-screened for swimming motility, biosurfactant production, and swarming motility. All candidates are listed in Table S1 (n =108) with the associated locus tag, gene name, GenBank description, KEGG BRITE Terms, and KEGG Pathway Terms (where applicable). The candidates were also grouped separately into hyper-swarm candidates (Table S2, n = 18) and swarm-deficient candidates (Table S3, n = 90). The swarm-deficient candidates were additionally grouped independently based on loss of biosurfactant production (Table S4, n = 25) or loss of swimming motility (Table S5, n = 82; note some of the candidates are listed in more than one table). The candidates in each table were categorized based on annotated function and are graphically summarized in Fig. 3.

### Loss of genes related to Gacamide A biosynthesis and secretion result in loss of swarming motility and changes in biosurfactant production

We identified a class of mutants defective for both swarming motility and biosurfactant production (Table S4) but swim proficiently. These mutants all had transposon insertions that mapped to a biosynthetic operon reported to encode the machinery needed to produce the non-ribosomal cyclic lipopeptide biosurfactant Gacamide A. Briefly, this machinery consists of modules containing a condensation, adenylation, and thiolation domain which iteratively add an amino acid to the growing peptide chain. The growing chain is then released and circularized by the tandem thioesterase domain encoded in *gamC* (45). Gacamide A can promote swarming motility when purified and exogenously supplemented to the swarm-deficient parental *P. fluorescens* Pf0-1 (34, 45, 46). The *gamA*, *gamB*, and *gamC* genes encode the biosynthetic proteins for Gacamide A. The *pleA*, *pleB*, and *pleC* genes are predicted to encode the Gacamide A secretion system given their sequence similarity to the MacAB-TolC type macrolide efflux pumps and conservation among cyclic lipopeptide-producing pseudomonads (45, 47–49).

Given these identified candidate genes, we conducted a genetic analysis utilizing the swarm-proficient Δ*rsmA* Δ*rsmE* Δ*rsmI* strain and introduced additional single chromosomal deletions of the predicted biosynthetic (*gamA*, *gamB*, and *gamC*) genes and transport (*pleA*, *pleB*, and *pleC*) genes. For these operonic genes, the individual deletion constructs were designed such that the relevant promoters remained intact to drive the expression of any downstream genes. These strains were then probed for biosurfactant production and swarm motility.

**Fig 4:**
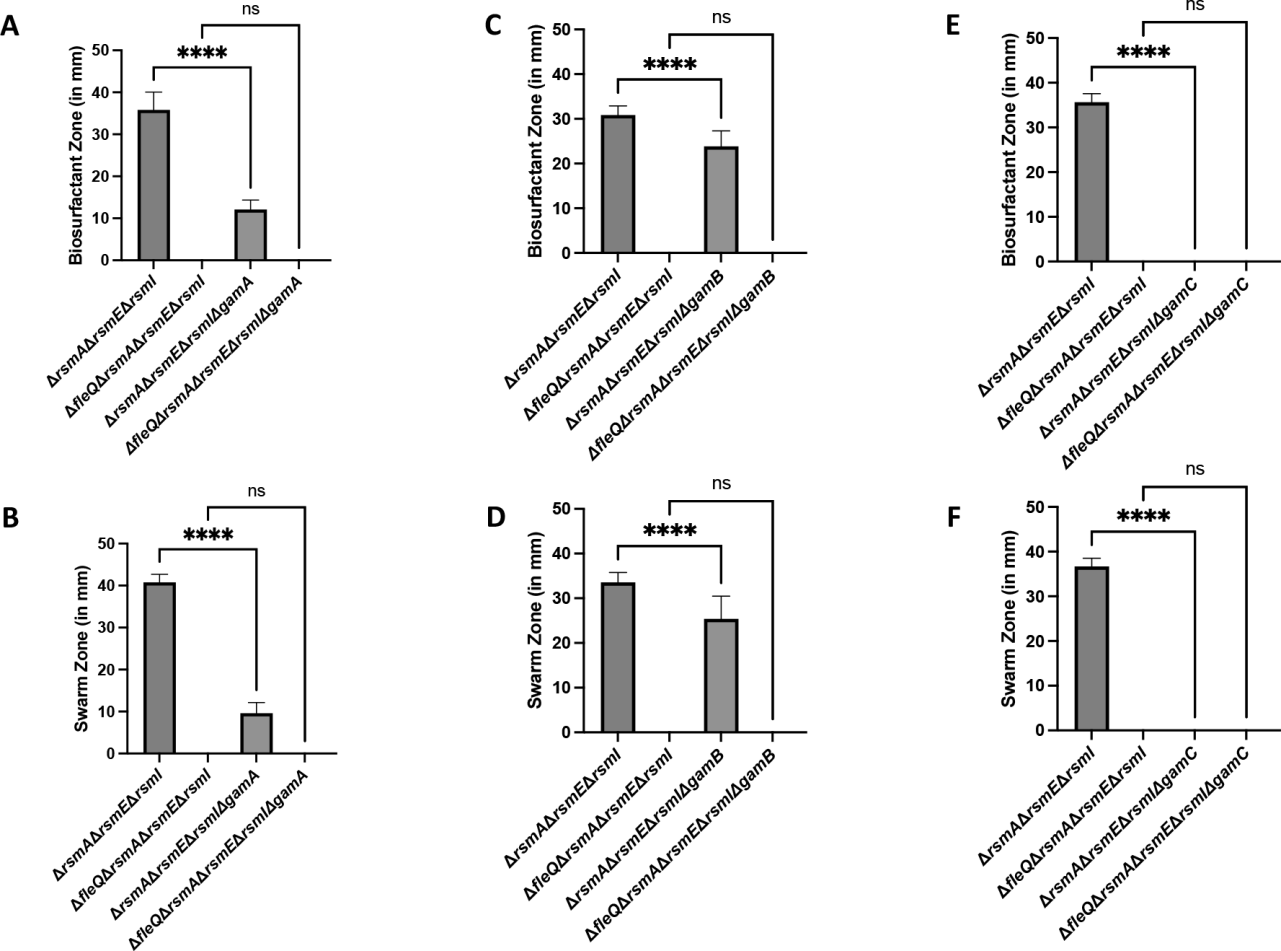
Loss of components of the biosurfactant operon effect swarming motility through the altered biosurfactant levels. (**A**) The biosurfactant zone (in millimeters) of the Δ*rsmA* Δ*rsmE* Δ*rsmI* triple mutant, Δ*fleQ* Δ*rsmA* Δ*rsmE* Δ*rsmI* quadruple mutant, Δ*rsmA* Δ*rsmE* Δ*rsmI* Δ*gamA* quadruple mutant, and the Δ*fleQ* Δ*rsmA* Δ*rsmE* Δ*rsmI* Δ*gamA* quintuple mutant after inoculation of 2.5 µL of overnight culture on the surface of KA minimal medium supplemented with 0.5% agar followed by 24-h growth at 30°C. (**B**) The swarm zone (in millimeters) of the Δ*rsmA* Δ*rsmE* Δ*rsmI* triple mutant, Δ*fleQ* Δ*rsmA* Δ*rsmE* Δ*rsmI* quadruple mutant, Δ*rsmA* Δ*rsmE* Δ*rsmI* Δ*gamA* quadruple mutant, and the Δ*fleQ* Δ*rsmA* Δ*rsmE* Δ*rsmI* Δ*gamA* quintuple mutant after inoculation of 2.5 µL of overnight culture on the surface of KA minimal medium supplemented with 0.5% agar followed by 24-h growth at 30°C and then 24-h growth at room temperature. (**C**) The biosurfactant zone (in millimeters) of the Δ*rsmA* Δ*rsmE* Δ*rsmI* triple mutant, Δ*fleQ* Δ*rsmA* Δ*rsmE* Δ*rsmI* quadruple mutant, Δ*rsmA* Δ*rsmE* Δ*rsmI* Δ*gamB* quadruple mutant, and the Δ*fleQ* Δ*rsmA* Δ*rsmE* Δ*rsmI* Δ*gamB* quintuple mutant after inoculation of 2.5 µL of overnight culture on the surface of KA minimal medium supplemented with 0.5% agar followed by 24-h growth at 30°C. (**D**) The swarm zone (in millimeters) of the Δ*rsmA* Δ*rsmE* Δ*rsmI* triple mutant, Δ*fleQ* Δ*rsmA* Δ*rsmE* Δ*rsmI* quadruple mutant, Δ*rsmA* Δ*rsmE* Δ*rsmI* Δ*gamB* quadruple mutant, and the Δ*fleQ* Δ*rsmA* Δ*rsmE* Δ*rsmI* Δ*gamB* quintuple mutant after inoculation of 2.5 µL of overnight culture on the surface of KA minimal medium supplemented with 0.5% agar followed by 24-h growth at 30°C and then 24-h growth at room temperature. (**E**) The biosurfactant zone (in millimeters) of the Δ*rsmA* Δ*rsmE* Δ*rsmI* triple mutant, Δ*fleQ* Δ*rsmA* Δ*rsmE* Δ*rsmI* quadruple mutant, Δ*rsmA* Δ*rsmE* Δ*rsmI* Δ*gamC* quadruple mutant, and the Δ*fleQ* Δ*rsmA* Δ*rsmE* Δ*rsmI* Δ*gamC* quintuple mutant after inoculation of 2.5 µL of overnight culture on the surface of KA minimal medium supplemented with 0.5% agar followed by 24-h growth at 30°C. (**F**) The swarm zone (in millimeters) of the Δ*rsmA* Δ*rsmE* Δ*rsmI* triple mutant, Δ*fleQ* Δ*rsmA* Δ*rsmE* Δ*rsmI* quadruple mutant, Δ*rsmA* Δ*rsmE* Δ*rsmI* Δ*gamC* quadruple mutant, and the Δ*fleQ* Δ*rsmA* Δ*rsmE* Δ*rsmI* Δ*gamC* quintuple mutant after inoculation of 2.5 µL of overnight culture on the surface of KA minimal medium supplemented with 0.5% agar followed by 24-h growth at 30°C and then 24-h growth at room temperature. Statistical significance for this figure was determined using one-way ANOVA with Tukey’s multiple comparisons tests. ****P < 0.0001. All error bars represent standard deviation.

**Fig 5:**
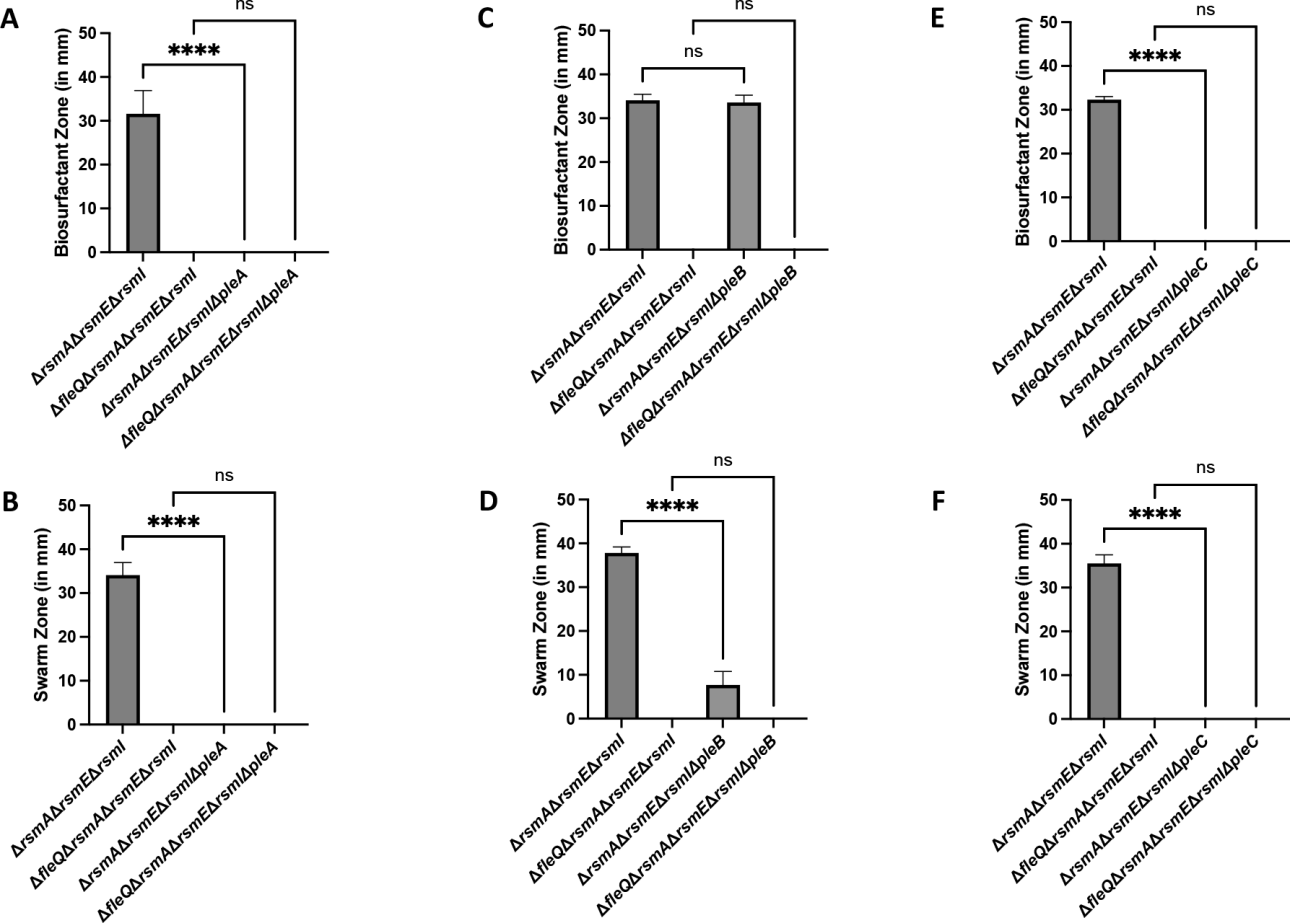
Loss of any component of the biosurfactant secretion system results in a swarm motility defect. (**A**) The biosurfactant zone (in millimeters) of the Δ*rsmA* Δ*rsmE* Δ*rsmI* triple mutant, Δ*fleQ* Δ*rsmA* Δ*rsmE* Δ*rsmI* quadruple mutant, Δ*rsmA* Δ*rsmE* Δ*rsmI* Δ*pleA* quadruple mutant, and the Δ*fleQ* Δ*rsmA* Δ*rsmE* Δ*rsmI* Δ*pleA* quintuple mutant after inoculation of 2.5 µL of overnight culture on the surface of KA minimal medium supplemented with 0.5% agar followed by 24-h growth at 30°C. (**B**) The swarm zone (in millimeters) of the Δ*rsmA* Δ*rsmE* Δ*rsmI* triple mutant, Δ*fleQ* Δ*rsmA* Δ*rsmE* Δ*rsmI* quadruple mutant, Δ*rsmA* Δ*rsmE* Δ*rsmI* Δ*pleA* quadruple mutant, and the Δ*fleQ* Δ*rsmA* Δ*rsmE* Δ*rsmI* Δ*pleA* quintuple mutant after inoculation of 2.5 µL of overnight culture on the surface of KA minimal medium supplemented with 0.5% followed by 24-h growth at 30°C and then 24-h growth at room temperature. (**C**) The biosurfactant zone (in millimeters) of the Δ*rsmA* Δ*rsmE* Δ*rsmI* triple mutant, Δ*fleQ* Δ*rsmA* Δ*rsmE* Δ*rsmI* quadruple mutant, Δ*rsmA* Δ*rsmE* Δ*rsmI* Δ*pleB* quadruple mutant, and the Δ*fleQ* Δ*rsmA* Δ*rsmE* Δ*rsmI* Δ*pleB* quintuple mutant after inoculation of 2.5 µL of overnight culture on the surface of KA minimal medium supplemented with 0.5% agar followed by 24-h growth at 30°C. (**D**) The swarm zone (in millimeters) of the Δ*rsmA* Δ*rsmE* Δ*rsmI* triple mutant, Δ*fleQ* Δ*rsmA* Δ*rsmE* Δ*rsmI* quadruple mutant, Δ*rsmA* Δ*rsmE* Δ*rsmI* Δ*pleB* quadruple mutant, and the Δ*fleQ* Δ*rsmA* Δ*rsmE* Δ*rsmI* Δ*pleB* quintuple mutant after inoculation of 2.5 µL of overnight culture on the surface of KA minimal medium supplemented with 0.5% agar followed by 24-h growth at 30°C and then 24-h growth at room temperature. (**E**) The biosurfactant zone (in millimeters) of the Δ*rsmA* Δ*rsmE* Δ*rsmI* triple mutant, Δ*fleQ* Δ*rsmA* Δ*rsmE* Δ*rsmI* quadruple mutant, Δ*rsmA* Δ*rsmE* Δ*rsmI* Δ*pleC* quadruple mutant, and the Δ*fleQ* Δ*rsmA* Δ*rsmE* Δ*rsmI* Δ*pleC* quintuple mutant after inoculation of 2.5 µL of overnight culture on the surface of KA minimal medium supplemented with 0.5% agar followed by 24-h growth at 30°C. (**F**) Swarm zone (in millimeters) of the Δ*rsmA* Δ*rsmE* Δ*rsmI* triple mutant, Δ*fleQ* Δ*rsmA* Δ*rsmE* Δ*rsmI* quadruple mutant, Δ*rsmA* Δ*rsmE* Δ*rsmI* Δ*pleC* quadruple mutant, and the Δ*fleQ* Δ*rsmA* Δ*rsmE* Δ*rsmI* Δ*pleC* quintuple mutant after inoculation of 2.5 µL of overnight culture on the surface of KA minimal medium supplemented with 0.5% agar followed by 24-h growth at 30°C and then 24-h growth at room temperature. Statistical significance was determined using one-way ANOVA with Tukey’s multiple comparisons tests. ****P < 0.0001. All error bars represent standard deviation.

For the genes predicted to encode functions required for Gacamide A production, deletion of *gamA* resulted in a marked decrease in biosurfactant production (Fig. 4A) and swarming motility (Fig. 4B), deletion of *gamB* resulted in a modest, but statistically significant decrease in biosurfactant production (Fig. 4C) and swarming motility (Fig. 4D), and deletion of *gamC* resulted in a complete loss of biosurfactant production (Fig. 4E) and swarming motility (Fig. 4F). Representative images of the swarm zones are included in Fig. S6. These results agree with previous work focused on *gamA* in a *P. fluorescens* Pf0-1 merodiploid strain containing both the native and *Pseudomonas protogens* Pf-5 *gacA* allele (34). For the genes predicted to encode functions required for Gacamide A export, loss of the predicted periplasmic or outer membrane components encoded by *pleA* and *pleC* gene, respectively, resulted in the complete loss of the biosurfactant zone (Fig. 5A and E, respectively) and swarming motility (Fig. 5B and F, respectively). Interestingly, loss of the predicted inner membrane component encoded by *pleB* did not result in a reduction of the biosurfactant zone (Fig. 5C) but did result in a sizeable decrease in swarming motility (Fig. 5D). Representative images of the swarm zones are included in Fig. S7.

To confirm that the observed differences in swarming motility were not related to changes in flagellar function, we assessed the strains for their ability to swim. After 24 h of growth at 30°C on swim agar, all tested mutants produced swim zones similar to their parental strain (Fig. S8 and S9). The similar swim zones also indicated that there was no general growth defect for any of these mutants.

### Loss of FliA results in a swarming and swimming motility defect

We identified several candidate mutants defective for both swimming and swarming motility (Table S5) but biosurfactant proficient. We focused our analysis specifically on the alternative sigma factor FliA, which was previously associated with flagellar biosynthesis and swarming motility regulation in *P. aeruginosa* (50, 51). We made a chromosomal deletion of *fliA* in the swarm-proficient Δ*rsmA* Δ*rsmE* Δ*rsmI* strain and assessed the mutant for the ability to swim and swarm.

**Fig 6:**
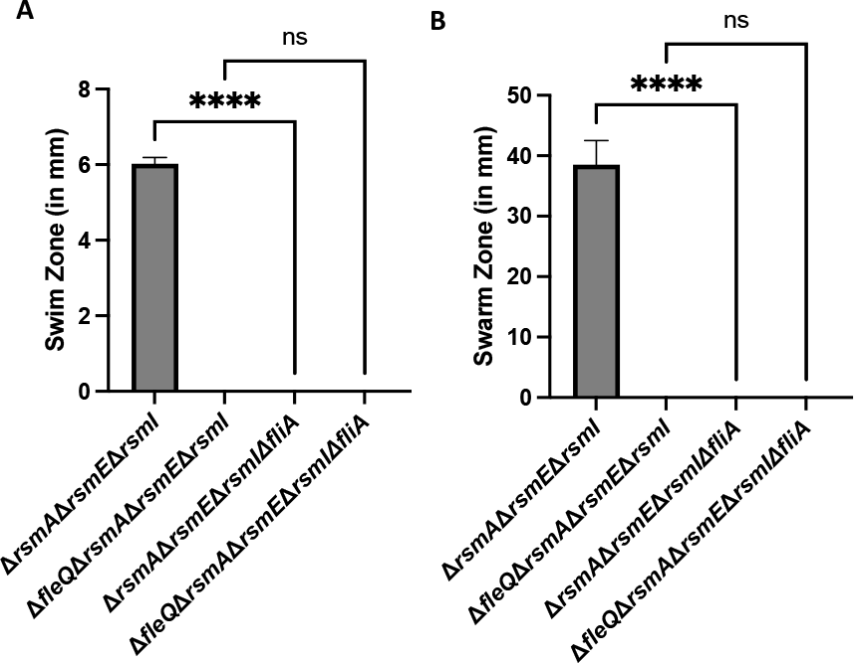
A FliA-deficient strain has a motility defect likely due to loss of flagellar function. (**A**) The swim zone (in millimeters) of the Δ*rsmA* Δ*rsmE* Δ*rsmI* triple mutant, Δ*fleQ* Δ*rsmA* Δ*rsmE* Δ*rsmI* quadruple mutant, Δ*rsmA* Δ*rsmE* Δ*rsmI* Δ*fliA* quadruple mutant, and the Δ*fleQ* Δ*rsmA* Δ*rsmE* Δ*rsmI* Δ*fliA* quintuple mutant after toothpick inoculation on KA minimal medium supplemented with 0.3% agar followed by 24-h growth at 30°C. (**B**) The swarm zone (in millimeters) of the Δ*rsmA* Δ*rsmE* Δ*rsmI* triple mutant, Δ*fleQ* Δ*rsmA* Δ*rsmE* Δ*rsmI* quadruple mutant, Δ*rsmA* Δ*rsmE* Δ*rsmI* Δ*fliA* quadruple mutant, and the Δ*fleQ* Δ*rsmA* Δ*rsmE* Δ*rsmI* Δ*fliA* quintuple mutant after inoculation of 2.5 µL of overnight culture on the surface of KA minimal medium supplemented with 0.5% agar followed by 24-h growth at 30°C and then 24-h growth at room temperature. Statistical significance for this figure was determined using one-way ANOVAs with Tukey’s multiple comparisons tests. ****P < 0.0001. All error bars represent standard deviation.

After 24 h of growth at 30°C on swim agar, the Δ*rsmA* Δ*rsmE* Δ*rsmI* Δ*fliA* strain was completely deficient in swimming motility (Fig. 6A). After 24 h of growth at 30°C and 24 h of growth at room temperature on swarm agar, the Δ*rsmA* Δ*rsmE* Δ*rsmI* Δ*fliA* strain was completely deficient in swarming motility (Fig. 6B). We noted no significant difference in biosurfactant production between the Δ*rsmA* Δ*rsmE* Δ*rsmI* Δ*fliA* mutant and its parental strain (Fig. S10), indicating that loss of FliA function results in loss of motility *via* loss of flagellar function.

## DISCUSSION

Our data here build upon the link between the Gac/Rsm pathway and swarming motility reported by our group and several other teams (22, 32, 34, 35, 42, 45, 46). Our genetic analyses demonstrate that loss of the Rsm proteins, which was previously shown to phenotypically mimic overstimulation of the Gac/Rsm pathway (36), induces biosurfactant production/secretion and swarming motility of *P. fluorescens* Pf0-1 and does so in a RsmA- and RsmE-dependent manner. Interestingly, previously published work demonstrated that loss of *rsmE* was sufficient to induce biosurfactant production on nutrient-rich Pseudomonas F Agar overlaid with polycarbonate membrane (46). However, this phenotype was not observed in this study when the *rsmE*-deficient strain was grown on swarm agar. We posit that these phenotypic differences are due to the media differences between these two studies, as Evans et al. optimized their conditions specifically for biosurfactant production (46), while the current study utilized a minimal, low-agar medium for swarming motility. Despite these differences, this current study demonstrates that overexpression of RsmE is sufficient to repress biosurfactant production and swarming motility. These findings support previously published data showing that a *P. fluorescens* Pf0-1 merodiploid strain containing the native Pf0-1 *gacA* gene and non-native *gacA* gene from *P. protogens* Pf-5 was proficient for swarming motility (34) and build on previous results demonstrating that RsmE regulates biosurfactant secretion by *P. fluorescens* Pf0-1 (46).

Interestingly, variation of the incubation profile suggests that biosurfactant deployment and swarming motility are both temperature-dependent processes. Contradictorily, biosurfactant deployment was optimal at 30°C while swarming motility was optimal at room temperature. These associations between temperature, motility, and biosurfactant deployment can be explored in future studies.

Our subsequent transposon mutagenesis identified numerous genes that contribute to swarming motility *via* the production of a functional flagellum, biosurfactant biosynthesis/secretion, or both. The mutants with a defect in biosurfactant production included strains with transposon insertions in genes encoding the non-ribosomal peptide synthetases responsible for synthesizing the biosurfactant Gacamide A and its predicted outer membrane transporter PleC. Subsequent genetic analyses revealed that loss of *gamA*, *gamB*, or *gamC* decreased biosurfactant secretion and swarming motility to varying degrees. These differences could be attributed to the likelihood that loss of the *gamA* or *gamB* gene still results in the production of basal levels of Gacamide A or an alternative surfactant that can partially support swarming motility. Evidence supporting this second idea has been previously reported by other groups, whereby domain deletions and substitutions within non-ribosomal peptide synthetases result in an alternative peptide being synthesized, which is a strategy currently being utilized to develop novel biotherapeutics (52–57). Based on the observed complete loss of swarming motility but the ability of this strain to swim, a mutation in the *gamC* gene likely results in a complete loss of biosurfactant production. The predicted terminal tandem thioesterase (TE-TE) domain of GamC is likely required for the circularization and release of the synthesized peptide from its peptidyl carrier protein (45, 58, 59). Interestingly, previously published mutations in *gamA via* antibiotic resistance cassette (34) or mini-Tn5 insertion (46) resulted in a complete loss of biosurfactant secretion, whereas a chromosomal deletion resulted in a sizeable decrease but not complete loss of biosurfactant secretion. We posit that disruption of the *gamA* gene *via* insertional mutagenesis has the unintended consequence of inactivating the whole operon, whereas chromosomal deletion likely allows for the continued production of the GamB and GamC proteins. With the loss of GamA *via* chromosomal deletion, it is likely that the peptide synthesis is initiated at the first condensation domain within the first module encoded in GamB resulting in a truncated variant of Gacamide A.

Genetic analysis revealed that the predicted Gacamide A secretion machinery is also required for swarming motility. As expected, loss of the periplasmic component PleA or the outer membrane component PleC of the proposed Gacamide A secretion machinery resulted in loss of detectable biosurfactant and complete loss of swarming motility. Interestingly, loss of the inner-membrane component PleB results in a complete loss of swarming motility even though this mutant strain is apparently proficient for biosurfactant production and swimming motility. These observations could suggest the presence of an alternative inner membrane transporter component that can substitute for PleB to support the secretion of Gacamide A, or that PleB may modify Gacamide A during secretion, which may be required for this surfactant to promote swarming motility.

The identified candidates with defects only in swimming and swarming motility clustered to a variety of seemingly unrelated pathways. Since multiple studies will be required to fully investigate the role of these many functions in motility, we focused here specifically on the alternative sigma factor FliA. As expected based on other studies in pseudomonads (50, 51, 60), a loss of FliA resulted in the loss of swimming and swarming motility, likely *via* loss of flagellar function.

Collectively, our findings provide evidence that swarming motility is regulated *via* the Gac/Rsm pathway in a RsmA- and RsmE-dependent manner by impacting flagellar motility and biosurfactant production/secretion. These findings provide a set of tools for the future study of these pathways*,* and we hope the findings from the transposon screen may be used by the broader community to study both motility and other Gac/Rsm-dependent phenotypes of *P. fluorescens* Pf0-1.

## MATERIALS AND METHODS

### Strains and media used in this study

*P. fluorescens* Pf0-1, *E. coli* S17-1 λ-pir, and *E. coli* SM10 λ-pir were used throughout this study. *E. coli* and *P. fluorescens* were routinely grown in lysogeny broth (LB) and *P. fluorescens* was routinely grown in KA minimal medium, as previously defined by Collins et al. (61). KA minimal medium contains 50 mM Tris-HCl (pH 7.4), 0.61 mM MgSO_4_, 1 mM K_2_HPO_4_, and 0.4% (wt/vol) L-arginine HCl. The medium was supplemented with 30 µg/mL gentamycin for *P. fluorescens* when harboring the expression vector pMQ72. For *E. coli*, the medium was supplemented with 10 ug/mL gentamycin when harboring the allelic exchange vector pMQ30 or the expression vector pMQ72, with 50 µg/mL carbenicillin for the strain harboring the transposon-containing shuttle vector pBT20, or 15 ug/mL tetracycline when harboring the allelic exchange plasmid pEX18Tc. The strains and plasmids used in this study are listed in Table S6.

### Construction of in-frame chromosomal gene deletions

For chromosomal deletions in this study, the allelic exchange vectors pEX18Tc or pMQ30 were utilized. Flanking regions of the target genes were amplified *via* PCR using Phusion polymerase (New England BioLabs). All primers used in the study are listed in Table S7.

The amplicons were integrated into SmaI (New England BioLabs)-digested vector using the GeneArt Gibson Assembly HiFi Master Mix (Invitrogen) according to the manufacturer’s specifications. Constructs were electroporated into *E. coli* S17-1 λ-pir and plated on LB agar with 15 µg/mL tetracycline or 10 µg/mL gentamycin for pEX18Tc or pMQ30-based constructs, respectively. Integration of the flanking regions into the vector was confirmed *via* PCR amplification and Sanger sequencing. Constructs were conjugated into *P. fluorescens*, whereby 1 mL aliquots of *E. coli* harboring the construct and *P. fluorescens* cultures grown overnight at 30°C were mixed in a 2 mL microcentrifuge tube, pelleted, washed in LB, and then plated on LB agar with no antibiotic selection to facilitate uptake of the deletion construct by *P. fluorescens*. After overnight incubation at 30°C, the cells were scraped from the plate and resuspended in fresh LB liquid medium. Serial dilutions were plated on LB agar supplemented with 45 µg/mL tetracycline and 30 µg/mL chloramphenicol for pEX18TC-based constructs or 30 µg/mL gentamycin and 30 µg/mL chloramphenicol for pMQ30-based constructs to select and counter select, respectively, for integration of the constructs into the *P. fluorescens* genome. To facilitate looping out of the drug resistance cassette, merodiploid candidates were initially grown in LB liquid without antibiotic selection overnight at 30°C and then serial dilutions were plated on LB agar without sodium chloride supplemented with 10% sucrose. Single colonies were struck on LB agar with and without antibiotic selection to confirm the loss of the resistance cassette carried by the plasmid. Antibiotic-susceptible candidates were screened for loss of the target gene *via* PCR amplification and Sanger sequencing.

### Construction of the *rsmA* expression vector

The *rsmA* gene was PCR amplified using Phusion polymerase (New England BioLabs) according to the manufacturer’s specifications. The primers were designed such that the forward primer contained the high-affinity T7 phage gene 10 ribosomal binding sites (62) and 8 bp spacer nucleotides upstream of the *rsmA* start codon. In addition, both primers contained 20 bp of homology to the pMQ72 SmaI cut-site at their 5′ ends. Primers used to build this construct are listed in Table S7.

The amplicon was integrated into SmaI-digested pMQ72 using the GeneArt Gibson Assembly HiFi Master Mix (Invitrogen) according to the manufacturer’s specifications. The construct was electroporated into *E. coli* S17-1 λ-pir, cells were recovered in LB for 1 h at 30°C and subsequently plated on LB agar supplemented with 10 µg/mL gentamycin. Antibiotic-resistant candidates were sequenced to confirm gene insertion into pMQ72. The construct was then electroporated into *P. fluorescens*, cells were initially recovered in LB for 1 h at 30°C, and dilutions were plated on LB agar supplemented with 30 µg/mL gentamycin to select for retention of the construct.

### Swim assay

Swim assays were conducted as previously defined in Pastora and O’Toole (36). Briefly, *P. fluorescens* Pf0-1 strains were grown overnight in 5 mL of LB with appropriate antibiotic selection at 30°C with agitation. 1 mL of aliquots was transferred to sterile 1.5 mL microcentrifuge tubes and used to stab inoculate KA minimal medium supplemented with 0.3% agar (swim agar). Inoculated plates were incubated for 24 h at 30°C. The diameter of the resulting swim zones was measured using a ruler.

### Biosurfactant and swarm assays

Biosurfactant and swarm assays were conducted using a modified approach previously described by Ha et al. (63). Briefly, *P. fluorescens* Pf0-1 strains were grown overnight in 5 mL of LB with appropriate antibiotic selection at 30°C with agitation. 2.5 µL of each culture was pipetted directly on the surface of KA minimal medium supplemented with 0.5% agar (swarm agar). Plates were initially incubated for 24 h at 30°C and the diameter of the resulting biosurfactant zones was measured using a ruler. Plates were then incubated for an additional 24 h at room temperature and the diameter of the resulting swarm zones was measured using a ruler.

### Transposon mutagenesis

Transposon mutagenesis was conducted as previously defined in Pastora and O’Toole (36). Briefly, *E. coli* harboring the pBT20 plasmid and the *P. fluorescens* Pf0-1 *ΔrsmAΔrsmEΔrsmI* triple mutant were grown in LB, with appropriate antibiotic selection, overnight at 30°C, and mixed in a 1:1 ratio in a 2 mL microcentrifuge tube. Cell pellets were then washed, resuspended, and plated on LB agar to facilitate conjugation of the pBT20 plasmid into *P. fluorescens*. Plates were incubated for 90 min at 30°C, and the resulting cells were scraped from the plate, washed in fresh LB, serially diluted, and plated on LB agar supplemented with 30 µg/mL gentamycin and 30 ug/mL chloramphenicol to select for transposon integration in *P. fluorescens*. Per Pastora and O’Toole (36), mutant libraries were generated by picking single colonies with sterile pipette tips into sterile 96-well flat-bottom polystyrene plates. Candidates were subsequently screened for their ability to swarm on swarm agar (see above). Candidates that were unable to swarm or were able to swarm at the 24-h timepoint and continue to swarm at the 48-h timepoint (hyper-swarmers) were identified from the mutant library. The identified candidates were struck out from the mutant library onto LB agar supplemented with 30 µg/mL gentamycin and subsequently re-tested for swarming motility to verify the swarm-deficient or hyper-swarm phenotype. Verified candidates were subsequently phenotypically screened for swimming motility and biosurfactant secretion. Transposon chromosomal insertion sites and transposon orientations were determined using arbitrary primed PCR as described by O’Toole et al. (64).

## Data Availability

This manuscript has no large datasets.

## SUPPLEMENTAL MATERIAL

Supplemental figures spectrum.00166-24-s0001.pdf Fig. S1 to S10.

Supplemental material spectrum.00166-24-s0002.pdf Tables S6 and S7.

Supplemental tables spectrum.00166-24-s0003.xlsx Tables S1 to S5.

ASM does not own the copyrights to Supplemental Material that may be linked to, or accessed through, an article. The authors have granted ASM a non-exclusive, world-wide license to publish the Supplemental Material files. Please contact the corresponding author directly for reuse.

## References

[1] Alberti L, Harshey RM. 1990. Differentiation of Serratia marcescens 274 into swimmer and swarmer cells. J Bacteriol 172:4322–4328. doi:10.1128/jb.172.8.4322-4328.1990 2198253 PMC213257

[2] O’Rear J, Alberti L, Harshey RM. 1992. Mutations that impair swarming motility in Serratia marcescens 274 include but are not limited to those affecting chemotaxis or flagellar function. J Bacteriol 174:6125–6137. doi:10.1128/jb.174.19.6125-6137.1992 1400161 PMC207679

[3] Harshey RM. 1994. Bees aren’t the only ones: swarming in Gram-negative bacteria. Mol Microbiol 13:389–394. doi:10.1111/j.1365-2958.1994.tb00433.x 7997156

[4] Matsuyama T, Bhasin A, Harshey RM. 1995. Mutational analysis of flagellum-independent surface spreading of Serratia marcescens 274 on a low-agar medium. J Bacteriol 177:987–991. doi:10.1128/jb.177.4.987-991.1995 7860610 PMC176693

[5] Mireles JR II, Toguchi A, Harshey RM. 2001. Salmonella enterica serovar Typhimurium swarming mutants with altered biofilm-forming abilities: surfactin inhibits biofilm formation . J Bacteriol 183:5848–5854. doi:10.1128/JB.183.20.5848-5854.2001 11566982 PMC99661

[6] Harshey RM. 2003. Bacterial motility on a surface: many ways to a common goal. Annu Rev Microbiol 57:249–273. doi:10.1146/annurev.micro.57.030502.091014 14527279

[7] Darnton NC, Turner L, Rojevsky S, Berg HC. 2010. Dynamics of bacterial swarming. Biophys J 98:2082–2090. doi:10.1016/j.bpj.2010.01.053 20483315 PMC2872219

[8] Berg HC. 2005. Swarming motility: it better be wet. Curr Biol 15:R599–R600. doi:10.1016/j.cub.2005.07.042 16085482

[9] Kearns DB, Losick R. 2003. Swarming motility in undomesticated Bacillus subtilis. Mol Microbiol 49:581–590. doi:10.1046/j.1365-2958.2003.03584.x 12864845

[10] Kearns DB. 2010. A field guide to bacterial swarming motility. Nat Rev Microbiol 8:634–644. doi:10.1038/nrmicro2405 20694026 PMC3135019

[11] Caiazza NC, Shanks RMQ, O’Toole GA. 2005. Rhamnolipids modulate swarming motility patterns of Pseudomonas aeruginosa. J Bacteriol 187:7351–7361. doi:10.1128/JB.187.21.7351-7361.2005 16237018 PMC1273001

[12] Caiazza NC, Merritt JH, Brothers KM, O’Toole GA. 2007. Inverse regulation of biofilm formation and swarming motility by Pseudomonas aeruginosa PA14. J Bacteriol 189:3603–3612. doi:10.1128/JB.01685-06 17337585 PMC1855903

[13] Kuchma SL, Griffin EF, O’Toole GA. 2012. Minor pilins of the type IV pilus system participate in the negative regulation of swarming motility. J Bacteriol 194:5388–5403. doi:10.1128/JB.00899-12 22865844 PMC3457191

[14] Köhler T, Curty LK, Barja F, van Delden C, Pechère JC. 2000. Swarming of Pseudomonas aeruginosa is dependent on cell-to-cell signaling and requires flagella and pili. J Bacteriol 182:5990–5996. doi:10.1128/JB.182.21.5990-5996.2000 11029417 PMC94731

[15] Anyan ME, Amiri A, Harvey CW, Tierra G, Morales-Soto N, Driscoll CM, Alber MS, Shrout JD. 2014. Type IV pili interactions promote intercellular association and moderate swarming of Pseudomonas aeruginosa. Proc Natl Acad Sci U S A 111:18013–18018. doi:10.1073/pnas.1414661111 25468980 PMC4273417

[16] Kuchma Sherry L, Brothers KM, Merritt JH, Liberati NT, Ausubel FM, O’Toole GA. 2007. BifA, a cyclic-di-GMP phosphodiesterase, inversely regulates biofilm formation and swarming motility by Pseudomonas aeruginosa PA14. J Bacteriol 189:8165–8178. doi:10.1128/JB.00586-07 17586641 PMC2168662

[17] Kuchma S L, Ballok AE, Merritt JH, Hammond JH, Lu W, Rabinowitz JD, O’Toole GA. 2010. Cyclic-di-GMP-mediated repression of swarming motility by Pseudomonas aeruginosa: the pilY1 gene and its impact on surface-associated behaviors. J Bacteriol 192:2950–2964. doi:10.1128/JB.01642-09 20233936 PMC2901681

[18] Baker AE, Diepold A, Kuchma SL, Scott JE, Ha DG, Orazi G, Armitage JP, O’Toole GA. 2016. A PilZ domain protein FlgZ mediates c-di-GMP-dependent swarming motility control in Pseudomonas aeruginosa. J Bacteriol 198:1837–1846. doi:10.1128/JB.00196-16 27114465 PMC4907108

[19] Luo Y, Zhao K, Baker AE, Kuchma SL, Coggan KA, Wolfgang MC, Wong GCL, O’Toole GA. 2015. A hierarchical cascade of second messengers regulates Pseudomonas aeruginosa surface behaviors. mBio 6:e02456-14. doi:10.1128/mBio.02456-14 25626906 PMC4324313

[20] Merritt JH, Brothers KM, Kuchma SL, O’Toole GA. 2007. SadC reciprocally influences biofilm formation and swarming motility via modulation of exopolysaccharide production and flagellar function. J Bacteriol 189:8154–8164. doi:10.1128/JB.00585-07 17586642 PMC2168701

[21] Shrout JD, Chopp DL, Just CL, Hentzer M, Givskov M, Parsek MR. 2006. The impact of quorum sensing and swarming motility on Pseudomonas aeruginosa biofilm formation is nutritionally conditional. Mol Microbiol 62:1264–1277. doi:10.1111/j.1365-2958.2006.05421.x 17059568

[22] Heurlier K, Williams F, Heeb S, Dormond C, Pessi G, Singer D, Cámara M, Williams P, Haas D. 2004. Positive control of swarming, rhamnolipid synthesis, and lipase production by the posttranscriptional RsmA/RsmZ system in Pseudomonas aeruginosa PAO1. J Bacteriol 186:2936–2945. doi:10.1128/JB.186.10.2936-2945.2004 15126453 PMC400603

[23] Hou L, Debru A, Chen Q, Bao Q, Li K. 2019. AmrZ regulates swarming motility through cyclic di-GMP-dependent motility inhibition and controlling Pel polysaccharide production in Pseudomonas aeruginosa PA14. Front Microbiol 10:1847. doi:10.3389/fmicb.2019.01847 31474950 PMC6707383

[24] Jiménez-Fernández A, López-Sánchez A, Jiménez-Díaz L, Navarrete B, Calero P, Platero AI, Govantes F. 2016. Complex interplay between FleQ, cyclic diguanylate and multiple σ factors coordinately regulates flagellar motility and biofilm development in Pseudomonas putida. PLoS One 11:e0163142. doi:10.1371/journal.pone.0163142 27636892 PMC5026340

[25] Yeung ATY, Torfs ECW, Jamshidi F, Bains M, Wiegand I, Hancock REW, Overhage J. 2009. Swarming of Pseudomonas aeruginosa is controlled by a broad spectrum of transcriptional regulators, including MetR. J Bacteriol 191:5592–5602. doi:10.1128/JB.00157-09 19592586 PMC2737960

[26] Hockett KL, Burch AY, Lindow SE. 2013. Thermo-regulation of genes mediating motility and plant interactions in Pseudomonas syringae. PLoS One 8:e59850. doi:10.1371/journal.pone.0059850 23527276 PMC3602303

[27] Nogales J, Vargas P, Farias GA, Olmedilla A, Sanjuán J, Gallegos M-T. 2015. FleQ coordinates flagellum-dependent and -independent motilities in Pseudomonas syringae pv. tomato DC3000. Appl Environ Microbiol 81:7533–7545. doi:10.1128/AEM.01798-15 26296726 PMC4592877

[28] Wu L, McGrane RS, Beattie GA. 2013. Light regulation of swarming motility in Pseudomonas syringae integrates signaling pathways mediated by a bacteriophytochrome and a LOV protein. mBio 4:e00334-13. doi:10.1128/mBio.00334-13 23760465 PMC3684834

[29] Barahona E, Navazo A, Martínez-Granero F, Zea-Bonilla T, Pérez-Jiménez RM, Martín M, Rivilla R. 2011. Pseudomonas fluorescens F113 mutant with enhanced competitive colonization ability and improved biocontrol activity against fungal root pathogens. Appl Environ Microbiol 77:5412–5419. doi:10.1128/AEM.00320-11 21685161 PMC3147442

[30] Simons M, van der Bij AJ, Brand I, de Weger LA, Wijffelman CA, Lugtenberg BJ. 1996. Gnotobiotic system for studying rhizosphere colonization by plant growth-promoting Pseudomonas bacteria. Mol Plant Microbe Interact 9:600–607. doi:10.1094/mpmi-9-0600 8810075

[31] de Weert S, Vermeiren H, Mulders IHM, Kuiper I, Hendrickx N, Bloemberg GV, Vanderleyden J, De Mot R, Lugtenberg BJJ. 2002. Flagella-driven chemotaxis towards exudate components is an important trait for tomato root colonization by Pseudomonas fluorescens. Mol Plant Microbe Interact 15:1173–1180. doi:10.1094/MPMI.2002.15.11.1173 12423023

[32] Sánchez-Contreras M, Martín M, Villacieros M, O’Gara F, Bonilla I, Rivilla R. 2002. Phenotypic selection and phase variation occur during alfalfa root colonization by Pseudomonas fluorescens F113. J Bacteriol 184:1587–1596. doi:10.1128/JB.184.6.1587-1596.2002 11872710 PMC134892

[33] Compeau G, Al-Achi BJ, Platsouka E, Levy SB. 1988. Survival of rifampin-resistant mutants of Pseudomonas fluorescens and Pseudomonas putida in soil systems. Appl Environ Microbiol 54:2432–2438. doi:10.1128/aem.54.10.2432-2438.1988 3144244 PMC204279

[34] Seaton SC, Silby MW, Levy SB. 2013. Pleiotropic effects of GacA on Pseudomonas fluorescens Pf0-1 in vitro and in soil. Appl Environ Microbiol 79:5405–5410. doi:10.1128/AEM.00819-13 23811507 PMC3753959

[35] Loper JE, Hassan KA, Mavrodi DV, Davis EW, Lim CK, Shaffer BT, Elbourne LDH, Stockwell VO, Hartney SL, Breakwell K, et al.. 2012. Comparative genomics of plant-associated Pseudomonas spp.: insights into diversity and inheritance of traits involved in multitrophic interactions. PLoS Genet 8:e1002784. doi:10.1371/journal.pgen.1002784 22792073 PMC3390384

[36] Pastora AB, O’Toole GA. 2023. The regulator FleQ both transcriptionally and post-transcriptionally regulates the level of RTX adhesins of Pseudomonas fluorescens J Bacteriol 205:e0015223. doi:10.1128/jb.00152-23 37655913 PMC10521353

[37] Lapouge K, Schubert M, Allain F-T, Haas D. 2008. Gac/Rsm signal transduction pathway of γ-proteobacteria: from RNA recognition to regulation of social behaviour. Mol Microbiol 67:241–253. doi:10.1111/j.1365-2958.2007.06042.x 18047567

[38] Ferreiro M-D, Gallegos M-T. 2021. Distinctive features of the Gac-Rsm pathway in plant-associated Pseudomonas. Environ Microbiol 23:5670–5689. doi:10.1111/1462-2920.15558 33939255

[39] Alsohim AS, Taylor TB, Barrett GA, Gallie J, Zhang X-X, Altamirano-Junqueira AE, Johnson LJ, Rainey PB, Jackson RW. 2014. The biosurfactant viscosin produced by Pseudomonas fluorescens SBW25 aids spreading motility and plant growth promotion. Environ Microbiol 16:2267–2281. doi:10.1111/1462-2920.12469 24684210

[40] Burch AY, Shimada BK, Mullin SWA, Dunlap CA, Bowman MJ, Lindow SE. 2012. Pseudomonas syringae coordinates production of a motility-enabling surfactant with flagellar assembly. J Bacteriol 194:1287–1298. doi:10.1128/JB.06058-11 22194459 PMC3294827

[41] Arora SK, Ritchings BW, Almira EC, Lory S, Ramphal R. 1997. A transcriptional activator, FleQ, regulates mucin adhesion and flagellar gene expression in Pseudomonas aeruginosa in a cascade manner. J Bacteriol 179:5574–5581. doi:10.1128/jb.179.17.5574-5581.1997 9287015 PMC179431

[42] Navazo A, Barahona E, Redondo-Nieto M, Martínez-Granero F, Rivilla R, Martín M. 2009. Three independent signalling pathways repress motility in Pseudomonas fluorescens F113. Microb Biotechnol 2:489–498. doi:10.1111/j.1751-7915.2009.00103.x 21255280 PMC3815909

[43] Robleto EA, López-Hernández I, Silby MW, Levy SB. 2003. Genetic analysis of the AdnA regulon in Pseudomonas fluorescens: nonessential role of flagella in adhesion to sand and biofilm formation. J Bacteriol 185:453–460. doi:10.1128/JB.185.2.453-460.2003 12511490 PMC145307

[44] Casaz P, Happel A, Keithan J, Read DL, Strain SR, Levy SB. 2001. The Pseudomonas fluorescens transcription activator AdnA is required for adhesion and motility. Microbiology (Reading) 147:355–361. doi:10.1099/00221287-147-2-355 11158352

[45] Jahanshah G, Yan Q, Gerhardt H, Pataj Z, Lämmerhofer M, Pianet I, Josten M, Sahl H-G, Silby MW, Loper JE, Gross H. 2019. Discovery of the cyclic lipopeptide gacamide a by genome mining and repair of the defective GacA regulator in Pseudomonas fluorescens Pf0-1. J Nat Prod 82:301–308. doi:10.1021/acs.jnatprod.8b00747 30666877

[46] Evans AF, Wells MK, Denk J, Mazza W, Santos R, Delprince A, Kim W. 2022. Spatial structure formation by RsmE-regulated extracellular secretions in Pseudomonas fluorescens Pf0-1. J Bacteriol 204:e0028522. doi:10.1128/jb.00285-22 36165622 PMC9578434

[47] Kobayashi N, Nishino K, Yamaguchi A. 2001. Novel macrolide-specific ABC-type efflux transporter in Escherichia coli. J Bacteriol 183:5639–5644. doi:10.1128/JB.183.19.5639-5644.2001 11544226 PMC95455

[48] Girard L, Geudens N, Pauwels B, Höfte M, Martins JC, De Mot R. 2022. Transporter gene-mediated typing for detection and genome mining of lipopeptide-producing Pseudomonas. Appl Environ Microbiol 88:e0186921. doi:10.1128/AEM.01869-21 34731056 PMC8788793

[49] Fitzpatrick AWP, Llabrés S, Neuberger A, Blaza JN, Bai X-C, Okada U, Murakami S, van Veen HW, Zachariae U, Scheres SHW, Luisi BF, Du D. 2017. Structure of the MacAB–TolC ABC-type tripartite multidrug efflux pump. Nat Microbiol 2:17070. doi:10.1038/nmicrobiol.2017.70 28504659 PMC5447821

[50] Starnbach MN, Lory S. 1992. The fliA (rpoF) gene of Pseudomonas aeruginosa encodes an alternative sigma factor required for flagellin synthesis. Mol Microbiol 6:459–469. doi:10.1111/j.1365-2958.1992.tb01490.x 1560774

[51] Lo Y-L, Shen L, Chang C-H, Bhuwan M, Chiu C-H, Chang H-Y. 2016. Regulation of motility and phenazine pigment production by FliA is cyclic-di-GMP dependent in Pseudomonas aeruginosa PAO1. PLoS ONE 11:e0155397. doi:10.1371/journal.pone.0155397 27175902 PMC4866697

[52] Thong WL, Zhang Y, Zhuo Y, Robins KJ, Fyans JK, Herbert AJ, Law BJC, Micklefield J. 2021. Gene editing enables rapid engineering of complex antibiotic assembly lines. Nat Commun 12:6872. doi:10.1038/s41467-021-27139-1 34824225 PMC8616955

[53] Bozhüyük KA, Micklefield J, Wilkinson B. 2019. Engineering enzymatic assembly lines to produce new antibiotics. Curr Opin Microbiol 51:88–96. doi:10.1016/j.mib.2019.10.007 31743841 PMC6908967

[54] Bozhüyük KAJ, Fleischhacker F, Linck A, Wesche F, Tietze A, Niesert C-P, Bode HB. 2018. De novo design and engineering of non-ribosomal peptide synthetases. Nat Chem 10:275–281. doi:10.1038/nchem.2890 29461518

[55] Bozhüyük KAJ, Linck A, Tietze A, Kranz J, Wesche F, Nowak S, Fleischhacker F, Shi Y-N, Grün P, Bode HB. 2019. Modification and de novo design of non-ribosomal peptide synthetases using specific assembly points within condensation domains. Nat Chem 11:653–661. doi:10.1038/s41557-019-0276-z 31182822

[56] Calcott MJ, Ackerley DF. 2014. Genetic manipulation of non-ribosomal peptide synthetases to generate novel bioactive peptide products. Biotechnol Lett 36:2407–2416. doi:10.1007/s10529-014-1642-y 25214216

[57] Winn M, Fyans JK, Zhuo Y, Micklefield J. 2016. Recent advances in engineering nonribosomal peptide assembly lines. Nat Prod Rep 33:317–347. doi:10.1039/c5np00099h 26699732

[58] Miller BR, Gulick AM. 2016. Structural biology of non-ribosomal peptide synthetases. Methods Mol Biol 1401:3–29. doi:10.1007/978-1-4939-3375-4_1 26831698 PMC4760355

[59] Roongsawang N, Washio K, Morikawa M. 2007. In vivo characterization of tandem C-terminal thioesterase domains in arthrofactin synthetase. Chembiochem 8:501–512. doi:10.1002/cbic.200600465 17328008

[60] Rodríguez-Herva JJ, Duque E, Molina-Henares MA, Navarro-Avilés G, Van Dillewijn P, De La Torre J, Molina-Henares AJ, La Campa AS, Ran FA, Segura A, Shingler V, Ramos J-L. 2010. Physiological and transcriptomic characterization of a fliA mutant of Pseudomonas putida KT2440. Environ Microbiol Rep 2:373–380. doi:10.1111/j.1758-2229.2009.00084.x 23766109

[61] Collins AJ, Pastora AB, Smith TJ, O’Toole GA. 2020. MapA, a second large RTX adhesin conserved across the pseudomonads, contributes to biofilm formation by Pseudomonas fluorescens. J Bacteriol 202:e00277-20. doi:10.1128/JB.00277-20 32631946 PMC7925077

[62] Olins PO, Devine CS, Rangwala SH, Kavka KS. 1988. The T7 phage gene 10 leader RNA, a ribosome-binding site that dramatically enhances the expression of foreign genes in Escherichia coli. Gene 73:227–235. doi:10.1016/0378-1119(88)90329-0 3072257

[63] Ha D-G, Kuchma SL, O’Toole GA. 2014. Plate-based assay for swarming motility in *Pseudomonas aeruginosa*, p 67–72. In Filloux A, Ramos JL (ed), Pseudomonas methods and protocols. Springer, New York. 10.1007/978-1-4939-0473-0_8 PMC9006052 24818898

[64] O’Toole GA, Kolter R. 1998. Initiation of biofilm formation in Pseudomonas fluorescens WCS365 proceeds via multiple, convergent signalling pathways: a genetic analysis. Mol Microbiol 28:449–461. doi:10.1046/j.1365-2958.1998.00797.x 9632250

