# Supplementary material for: Multiple pathways impact the swarming motility of Pseudomonas fluorescens Pf0-1
Source: Microbiol Spectr. 2024 Apr 30;12(6):e00166-24. doi: 10.1128/spectrum.00166-24 (PMC11237744; doi:10.1128/spectrum.00166-24)
Supplement: Supplemental figures — Fig. S1 to S10. [file spectrum.00166-24-s0001.pdf]

Supplemental Figures

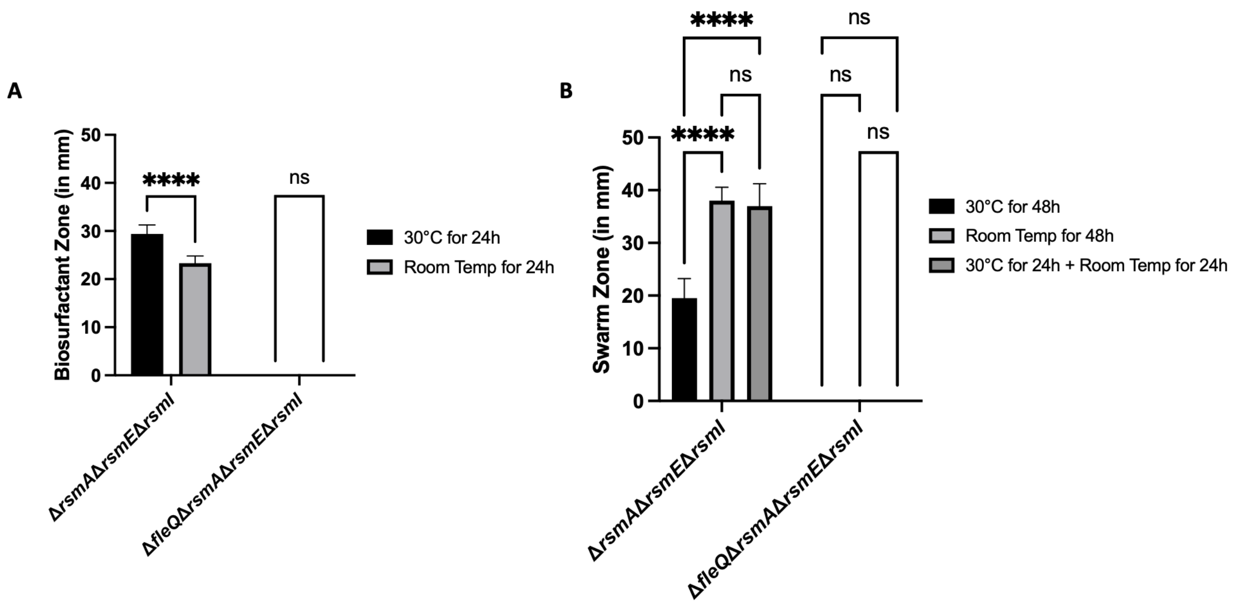

FIG S1 Biosurfactant deployment and swarming are temperature-dependent processes. (A) Biosurfactant zone (in millimeters) of the Δ*rsmA* Δ*rsmE* Δ*rsmI* triple mutant and Δ*fleQ* Δ<u>*rsmA*</u> Δr*smE* Δ*rsmI* quadruple mutant after inoculation of 2.5µl of overnight culture on the surface of KA minimal medium supplemented with 0.5% agar and incubated for 24h at 30°C or 24h at room temperature. (B) Swarm zone (in millimeters) of the Δ*rsmA* Δ*rsmE* Δ*rsmI* triple mutant and Δ*fleQ* Δ<u>*rsmA*</u> Δr*smE* Δ*rsmI* quadruple mutant after inoculation of 2.5µl of overnight culture on the surface of KA minimal medium supplemented with 0.5% agar and incubated for 48h at 30°C, 48h at room temperature, or 24h growth at 30°C then 24h growth at room temperature. statistical significance for this figure was determined using two-way ANOVAs with Tukey's multiple comparisons tests. ****, P<0.0001. All error bars represent standard deviation.

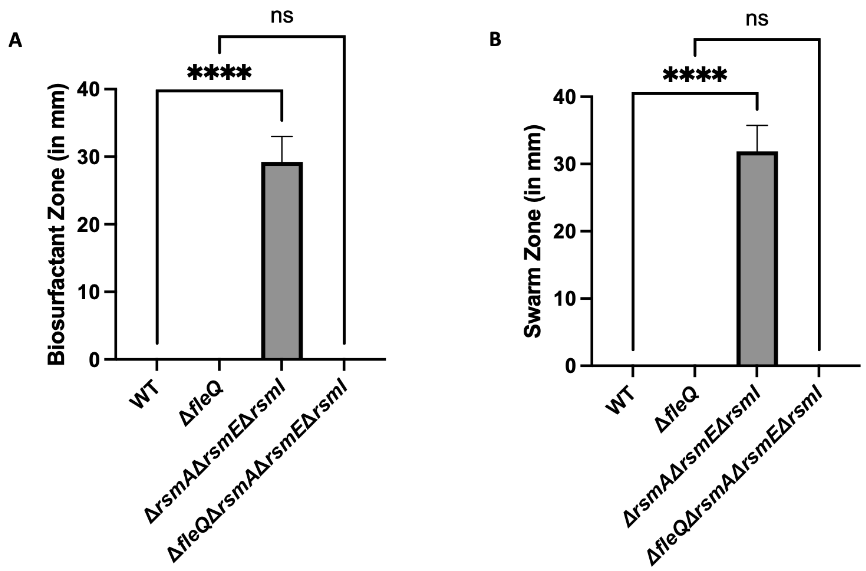

FIG S2 A strain deficient for the Rsm proteins swarms on 0.3% Agar. (A) Biosurfactant zone (in millimeters) of the WT strain, Δ*fleQ* single mutant, Δ*rsmA* Δ*rsmE* Δ*rsmI* triple mutant, and a Δ*fleQ* Δ*rsmA* Δr*smE* Δ*rsmI* quadruple mutant after inoculation of 2.5µl of overnight culture on the surface of KA minimal medium supplemented with 0.3% agar (swim agar) and incubated for 24h at 30°C. (B) Swarm zone (in millimeters) of the WT strain, Δ*fleQ* single mutant, Δ*rsmA* Δ*rsmE* Δ*rsmI* triple mutant, and a Δ*fleQ* Δ*rsmA* Δ*rsmE* Δ*rsmI* quadruple mutant after inoculation of 2.5µl of overnight culture on the surface of KA minimal medium supplemented with 0.3% agar (swim agar) followed by 24h growth at 30°C followed by 24h growth at room temperature. Statistical significance for this figure was determined using one-way ANOVA with Tukey's multiple comparisons tests. ****, P<0.0001. All error bars represent standard deviation.

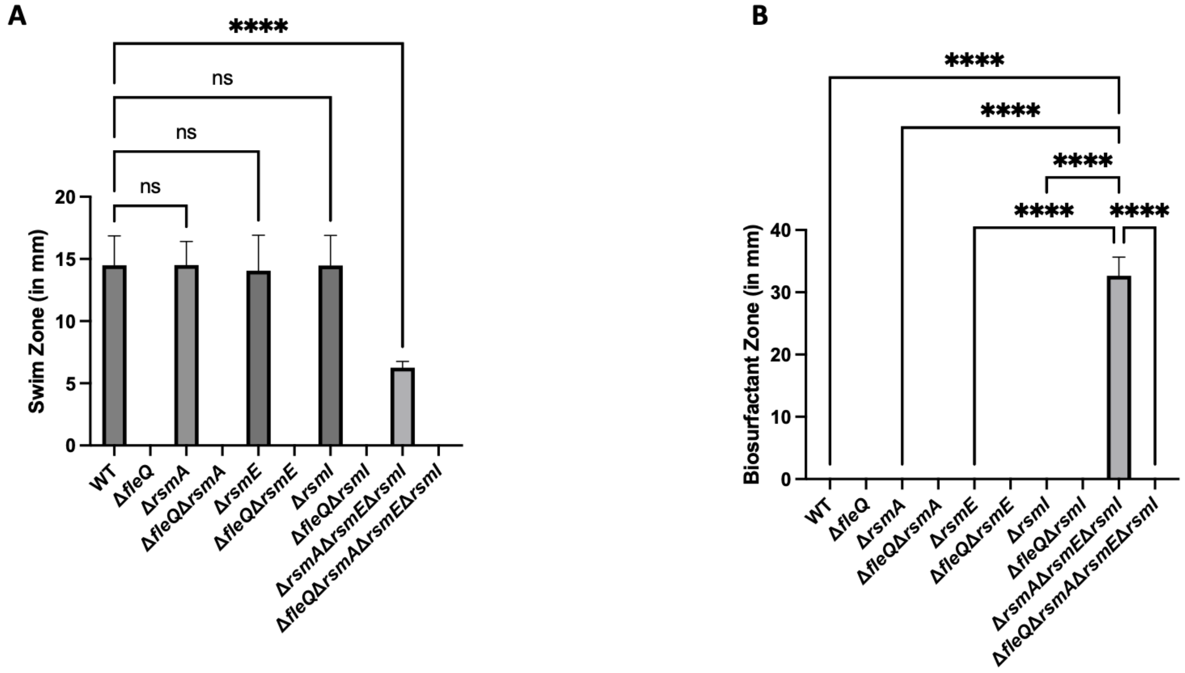

FIG S3 Loss of a single Rsm Protein is not sufficient for biosurfactant production. (A) Swim zone (in millimeters) of the WT strain, Δ*fleQ* single mutant, Δ*rsmA* single mutant, Δ*fleQ* Δ*rsmA* double mutant, Δ*rsmE* single mutant, Δ*fleQ* Δ*rsmE* double mutant, Δ*rsmI* single mutant, Δ*fleQ* Δ*rsmI* double mutant, Δ*rsmA* Δ*rsmE* Δ*rsmI* triple mutant, and a Δ*fleQ* Δ*rsmA* Δ*rsmE* Δ*rsmI* quadruple mutant after toothpick inoculation on KA minimal medium supplemented with 0.3% agar and 24h growth at 30°C. (B) Biosurfactant zone (in millimeters) of the WT strain, Δ*fleQ* single mutant, Δ*rsmA* single mutant, Δ*fleQ* Δ*rsmA* double mutant, Δ*rsmE* single mutant, Δ*fleQ* Δ*rsmE* double mutant, ΔrsmI single mutant, Δ*fleQ* Δ*rsmI* double mutant, Δ*rsmA* Δ*rsmE* Δ*rsmI* triple mutant, and a Δ*fleQ* Δ*rsmA* Δ*rsmE* Δ*rsmI* quadruple mutant after inoculation of 2.5µl of overnight culture on the surface of KA minimal medium supplemented with 0.5% agar and 24h growth at 30°C. Statistical significance for this figure was determined using one-way ANOVA with Tukey's multiple comparisons tests. ****, $P<0.0001$. All error bars represent standard deviation.

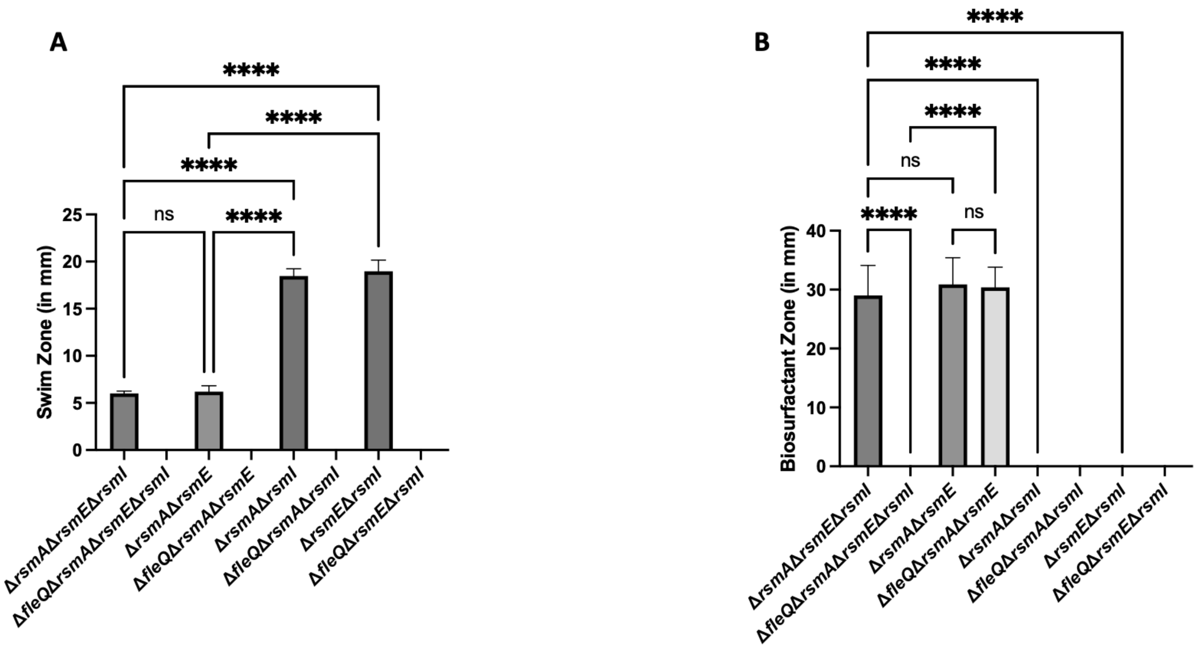

FIG S4 A strain deficient for both RsmA and RsmE is sufficient for biosurfactant production. (A) Swim zone (in millimeters) of the Δ*rsmA* Δ*rsmE* Δ*rsmI* triple mutant, Δ*fleQ* Δ*rsmA* Δ*rsmE* Δ*rsmI* quadruple mutant, Δ*rsmA* Δ*rsmE* double mutant, Δ*fleQ* Δ*rsmA* Δ*rsmE* triple mutant, Δ*rsmA* Δ*rsmI* double mutant, Δ*fleQ* Δ*rsmA* Δ*rsmI* triple mutant, Δ*rsmE* Δ*rsmI* double mutant, and Δ*fleQ* Δ*rsmE* Δ*rsmI* triple mutant after toothpick inoculation on KA minimal medium supplemented with 0.3% agar and 24h growth at 30°C. (B) Biosurfactant zone (in millimeters) the Δ*rsmA* Δ*rsmE* Δ*rsmI* triple mutant, Δ*fleQ* Δ*rsmA* Δ*rsmE* Δ*rsmI* quadruple mutant, ΔrsmA ΔrsmE double mutant, ΔfleQ ΔrsmA ΔrsmE triple mutant, ΔrsmA ΔrsmI double mutant, Δ*fleQ* Δ*rsmA* Δ*rsmI* triple mutant, Δ*rsmE* Δ*rsmI* double mutant, and Δ*fleQ* Δ*rsmE* Δ*rsmI* triple mutant after inoculation of 2.5µl of overnight culture on the surface of KA minimal medium supplemented with 0.5% agar and 24h growth at 30°C. Statistical significance for this figure was determined using one-way ANOVA with Tukey's multiple comparisons tests. ****, P<0.0001. All error bars represent standard deviation.

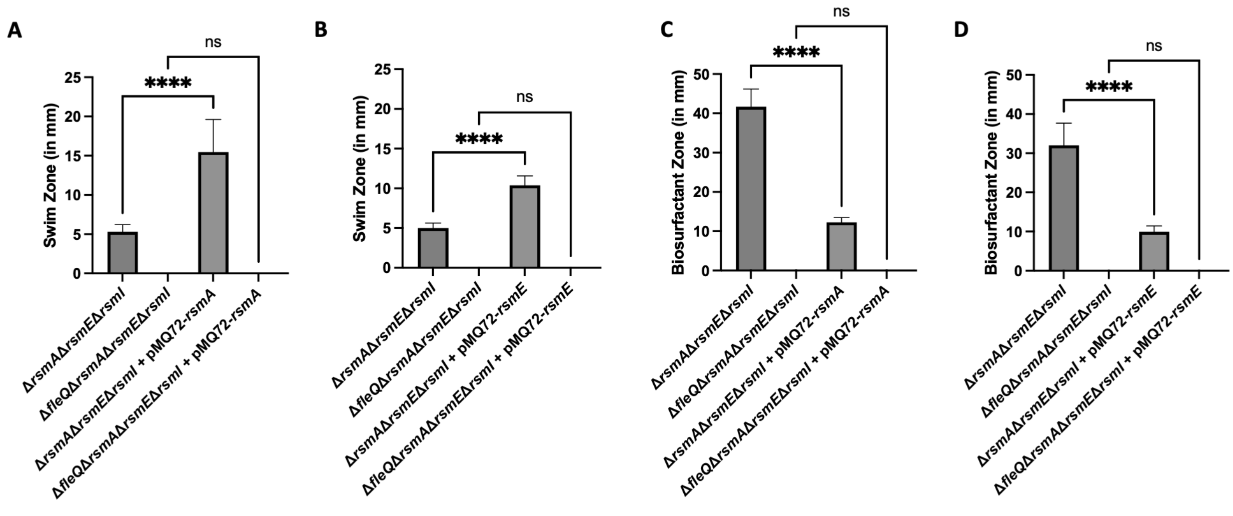

FIG S5 Expression of RsmA or RsmE from a plasmid reduces biosurfactant production in a strain deficient of the Rsm proteins. (A) Swim zone (in millimeters) of the Δ*rsmA* Δ*rsmE* Δ*rsmI* mutant, Δ*fleQ* Δ*rsmA* Δ*rsmE* Δ*rsmI* mutant, Δ*rsmA* Δ*rsmE* Δ*rsmI* mutant + pMQ72-*rsmA*, and Δ*fleQ* Δ*rsmA* Δ*rsmE* Δ*rsmI* + pMQ72-*rsmA* mutant after toothpick inoculation on KA minimal medium supplemented with 0.3% agar and 24h growth at 30°C. (B) Swim zone (in millimeters) of the Δ*rsmA* Δ*rsmE* Δ*rsmI* mutant, Δ*fleQ* Δ*rsmA* Δ*rsmE* Δ*rsmI* mutant, Δ*rsmA* Δ*rsmE* Δ*rsmI* mutant + pMQ72-*rsmE*, and Δ*fleQ* Δ*rsmA* Δ*rsmE* Δ*rsmI* + pMQ72-*rsmE* mutant after toothpick inoculation on KA minimal medium supplemented with 0.3% agar and 24h growth at 30°C. (C) Biosurfactant zone (in millimeters) of the Δ*rsmA* Δ*rsmE* Δ*rsmI* mutant, Δ*fleQ* Δ*rsmA* Δ*rsmE* Δ*rsmI* mutant, Δ*rsmA* Δ*rsmE* Δ*rsmI* mutant + pMQ72-*rsmA*, and Δ*fleQ* Δ*rsmA* Δ*rsmE* Δ*rsmI* + pMQ72-*rsmA* mutant after inoculation of 2.5µl of overnight culture on the surface of KA minimal medium supplemented with 0.5% agar and 24h growth at 30°C. (D) Biosurfactant zone (in millimeters) of the Δ*rsmA* Δ*rsmE* Δ*rsmI* mutant, Δ*fleQ* Δ*rsmA* Δ*rsmE* Δ*rsmI* mutant, Δ*rsmA* Δ*rsmE* Δ*rsmI* mutant + pMQ72-*rsmE*, and Δ*fleQ* Δ*rsmA* Δ*rsmE* Δ*rsmI* + pMQ72-*rsmE* mutant after inoculation of 2.5µl of overnight culture on the surface of KA minimal medium supplemented with 0.5% agar and 24h growth at 30°C. Statistical significance for this figure was determined using one-way ANOVA with Tukey's multiple comparisons tests. ****, P<0.0001. All error bars represent standard deviation.

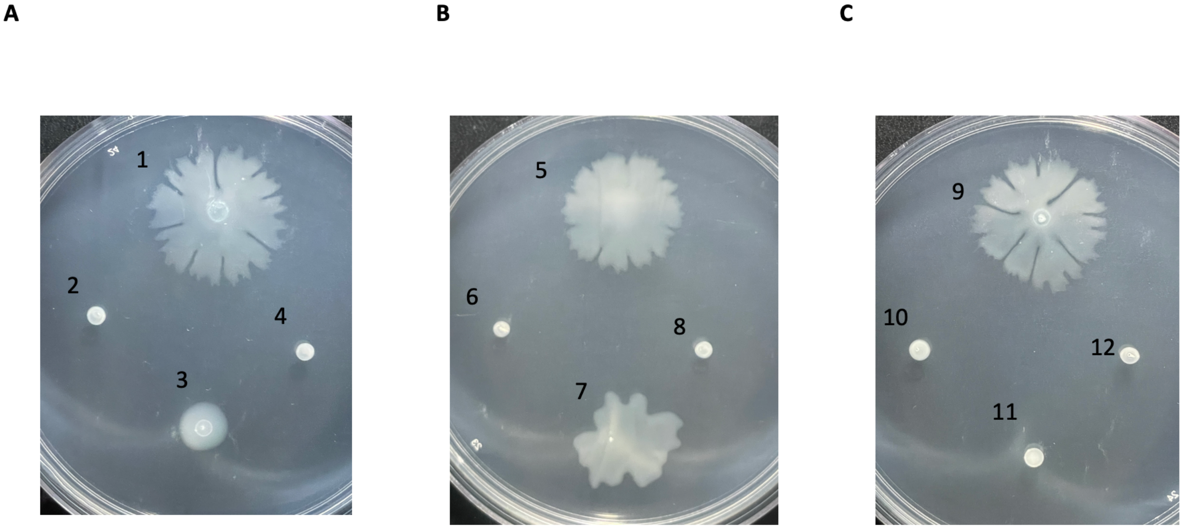

Fig S6 Loss of components of the operon coding for biosurfactant production effect swarming motility through the altered biosurfactant levels. (A) Representative image of swarm zones of the (1) Δ*rsmA* Δ*rsmE* Δ*rsmI* triple mutant, (2) Δ*fleQ* Δ*rsmA* Δ*rsmE* Δ*rsmI* quadruple mutant, (3) Δ*rsmA* Δ*rsmE* Δ*rsmI* Δ*gamA* quadruple mutant, and the (4) Δ*fleQ* Δ*rsmA* Δ*rsmE* Δ*rsmI* Δ*gamA* quintuple mutant after inoculation of 2.5µl of overnight culture on the surface of KA minimal medium supplemented with 0.5% agar followed by 24h growth at 30°C then 24h growth at room temperature. (B) Representative image of swarm zones of the (5) Δ*rsmA* Δ*rsmE* Δ*rsmI* triple mutant, (6) Δ*fleQ* Δ*rsmA* Δ*rsmE* Δ*rsmI* quadruple mutant, (7) Δ*rsmA* Δ*rsmE* Δ*rsmI* Δ*gamB* quadruple mutant, and the (8) Δ*fleQ* Δ*rsmA* Δ*rsmE* Δ*rsmI* Δ*gamB* quintuple mutant after inoculation of 2.5µl of overnight culture on the surface of KA minimal medium supplemented with 0.5% agar followed by 24h growth at 30°C then 24h growth at room temperature. (C) Representative image of swarm zones of the (9) Δ*rsmA* Δ*rsmE* Δ*rsmI* triple mutant, (10) Δ*fleQ* Δ*rsmA* Δ*rsmE* Δ*rsmI* quadruple mutant, (11) Δ*rsmA* Δ*rsmE* Δ*rsmI* Δ*gamC* quadruple mutant, and the (12) Δ*fleQ* Δ*rsmA* Δ*rsmE* Δ*rsmI* Δ*gamC* quintuple mutant after inoculation of 2.5µl of overnight culture on the surface of KA minimal medium supplemented with 0.5% agar followed by 24h growth at 30°C then 24h growth at room temperature.

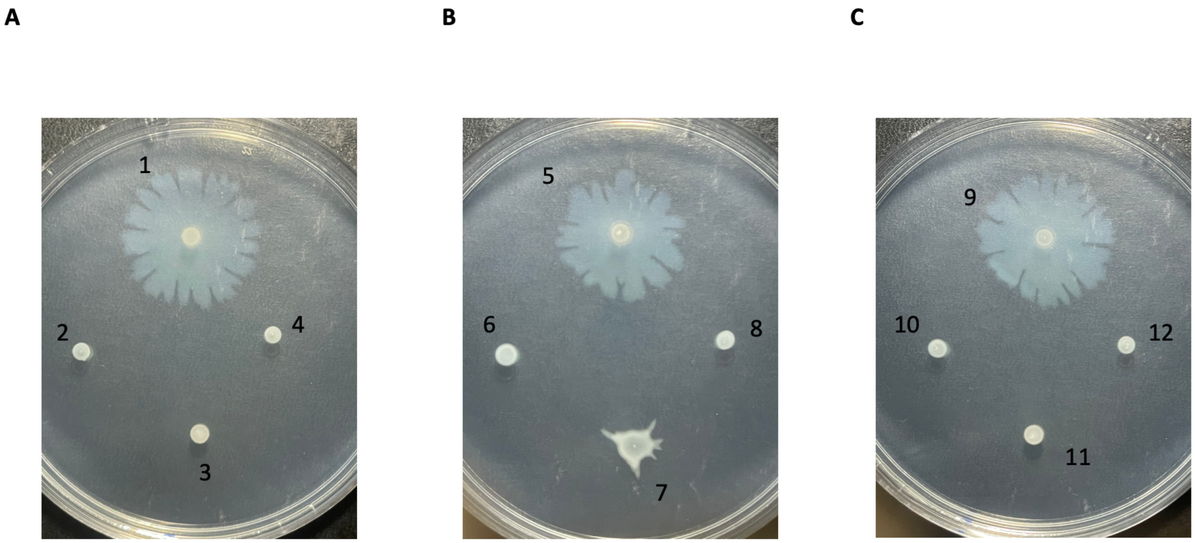

Fig S7 Loss of any component of the biosurfactant secretion system results in a swarm motility defect. (A) Representative image of swarm zones of the (1) Δ*rsmA* Δ*rsmE* Δ*rsmI* triple mutant, (2) Δ*fleQ* Δ*rsmA* Δ*rsmE* Δ*rsmI* quadruple mutant, (3) Δ*rsmA* Δ*rsmE* Δ*rsmI* Δ*pleA* quadruple mutant, and the (4) Δ*fleQ* Δ*rsmA* Δ*rsmE* Δ*rsmI* Δ*pleA* quintuple mutant after inoculation of 2.5µl of overnight culture on the surface of KA minimal medium supplemented with 0.5% agar followed by 24h growth at 30°C then 24h growth at room temperature. (B) Representative image of swarm zones of the (5) Δ*rsmA* Δ*rsmE* Δ*rsmI* triple mutant, (6) Δ*fleQ* Δ*rsmA* Δ*rsmE* Δ*rsmI* quadruple mutant, (7) Δ*rsmA* Δ*rsmE* Δ*rsmI* Δ*pleB* quadruple mutant, and the (8) Δ*fleQ* Δ*rsmA* Δ*rsmE* Δ*rsmI* Δ*pleB* quintuple mutant after inoculation of 2.5µl of overnight culture on the surface of KA minimal medium supplemented with 0.5% agar followed by 24h growth at 30°C then 24h growth at room temperature. (C) Representative image of swarm zones of the (9) Δ*rsmA* Δ*rsmE* Δ*rsmI* triple mutant, (10) Δ*fleQ* Δ*rsmA* Δ*rsmE* Δ*rsmI* quadruple mutant, (11) Δ*rsmA* Δ*rsmE* Δ*rsmI* Δ*pleC* quadruple mutant, and the (12) Δ*fleQ* Δ*rsmA* Δ*rsmE* Δ*rsmI* Δ*pleC* quintuple mutant after inoculation of 2.5µl of overnight culture on the surface of KA minimal medium supplemented with 0.5% agar followed by 24h growth at 30°C then 24h growth at room temperature.

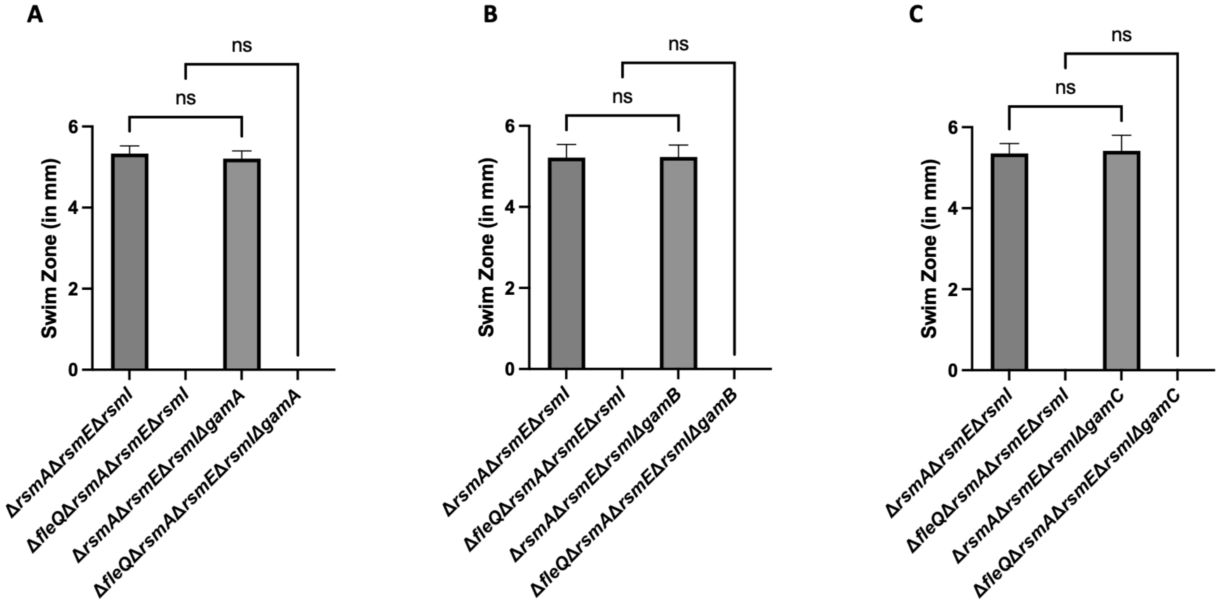

FIG S8 Loss of any component of the predicted biosurfactant biosynthetic machinery does not affect flagellar function as assessed by swimming motility. (A) Swim zone (in millimeters) of the Δ*rsmA* Δ*rsmE* Δ*rsmI* triple mutant, Δ*fleQ* Δ*rsmA* Δ*rsmE* Δ*rsmI* quadruple mutant, Δ*rsmA* Δ*rsmE* Δ*rsmI* Δ*gamA* quadruple mutant, and the Δ*fleQ* Δ*rsmA* Δ*rsmE* Δ*rsmI* Δ*gamA* quintuple mutant after toothpick inoculation on KA minimal medium supplemented with 0.3% agar and 24h growth at 30°C. (B) Swim zone (in millimeters) of the Δ*rsmA* Δ*rsmE* Δ*rsmI* triple mutant, Δ*fleQ* Δ*rsmA* Δ*rsmE* Δ*rsmI* quadruple mutant, Δ*rsmA* Δ*rsmE* Δ*rsmI* Δ*gamB* quadruple mutant, and the Δ*fleQ* Δ*rsmA* Δ*rsmE* Δ*rsmI* Δ*gamB* quintuple mutant after toothpick inoculation on KA minimal medium supplemented with 0.3% agar and 24h growth at 30°C. (C) Swim zone (in millimeters) of the Δ*rsmA* Δ*rsmE* Δ*rsmI* triple mutant, Δ*fleQ* Δ*rsmA* Δ*rsmE* Δ*rsmI* quadruple mutant, Δ*rsmA* Δ*rsmE* Δ*rsmI* Δ*gamC* quadruple mutant, and the Δ*fleQ* Δ*rsmA* Δ*rsmE* Δ*rsmI* Δ*gamC* quintuple mutant after toothpick inoculation on KA minimal medium supplemented with 0.3% agar and 24h growth at 30°C. Statistical significance for this figure was determined using one-way ANOVA with Tukey's multiple comparisons tests. All error bars represent standard deviation.

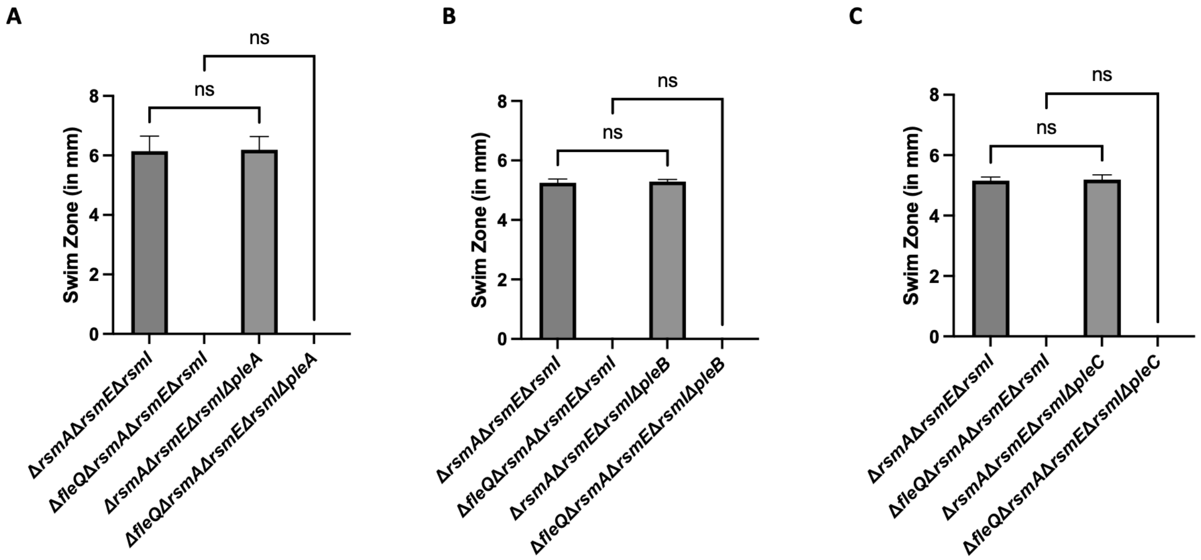

FIG S9 Loss of any component of the predicted biosurfactant secretion machinery does not affect flagellar function as assessed by swimming motility. (A) Swim zone (in millimeters) of the Δ*rsmA* Δ*rsmE* Δ*rsmI* triple mutant, Δ*fleQ* Δ*rsmA* Δ*rsmE* Δ*rsmI* quadruple mutant, Δ*rsmA* Δ*rsmE* Δ*rsmI* Δ*pleA* quadruple mutant, and the Δ*fleQ* Δ*rsmA* Δ*rsmE* Δ*rsmI* Δ*pleA* quintuple mutant after toothpick inoculation on KA minimal medium supplemented with 0.3% agar and 24h growth at 30°C. (B) Swim zone (in millimeters) of the Δ*rsmA* Δ*rsmE* Δ*rsmI* triple mutant, Δ*fleQ* Δ*rsmA* Δ*rsmE* Δ*rsmI* quadruple mutant, Δ*rsmA* Δ*rsmE* Δ*rsmI* Δ*pleB* quadruple mutant, and the Δ*fleQ* Δ*rsmA* Δ*rsmE* Δ*rsmI* Δ*pleB* quintuple mutant after toothpick inoculation on KA minimal medium supplemented with 0.3% agar and 24h growth at 30°C. (C) Swim zone (in millimeters) of the Δ*rsmA* Δ*rsmE* Δ*rsmI* triple mutant, Δ*fleQ* Δ*rsmA* Δ*rsmE* Δ*rsmI* quadruple mutant, Δ*rsmA* Δ*rsmE* Δ*rsmI* Δ*pleC* quadruple mutant, and the Δ*fleQ* Δ*rsmA* Δ*rsmE* Δ*rsmI* Δ*pleC* quintuple mutant after toothpick inoculation on KA minimal medium supplemented with 0.3% agar and 24h growth at 30°C. Statistical significance for this figure was determined using one-way ANOVA with Tukey's multiple comparisons tests. All error bars represent standard deviation.

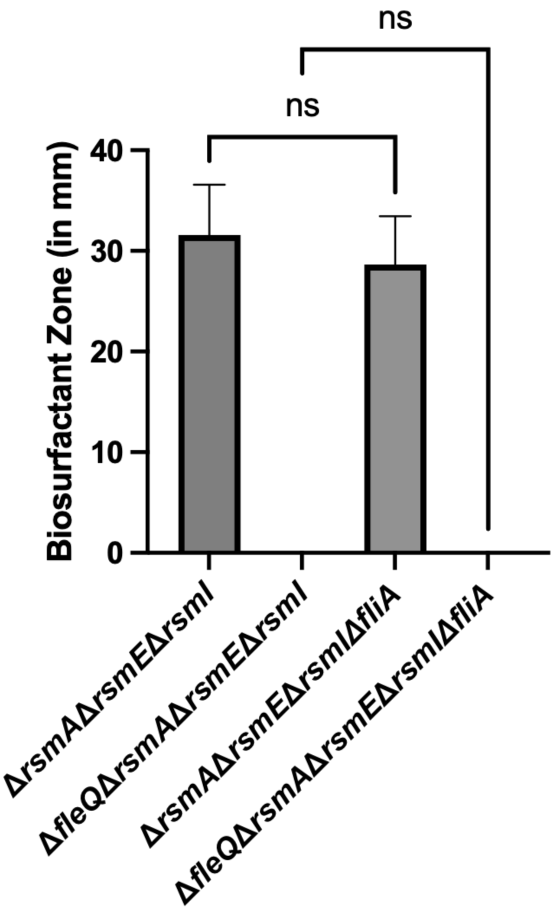

FIG S10 Loss of FliA has no impact on biosurfactant production. Biosurfactant zone (in millimeters) of the Δ*rsmA* Δ*rsmE* Δ*rsmI* triple mutant, Δ*fleQ* Δ*rsmA* Δ*rsmE* Δ*rsmI* quadruple mutant, Δ*rsmA* Δ*rsmE* Δ*rsmI* Δ*fliA* quadruple mutant, and the Δ*fleQ* Δ*rsmA* Δ*rsmE* Δ*rsmI* Δ*fliA* quintuple mutant after inoculation of 2.5µl of overnight culture on the surface of KA minimal medium supplemented with 0.5% agar and 24h growth at 30°C. Statistical significance was determined using a one-way ANOVA with Tukey's multiple comparisons tests. All error bars represent standard deviation.
