# Supplementary material for: Multiple pathways impact the swarming motility of Pseudomonas fluorescens Pf0-1
Source: Microbiol Spectr. 2024 Apr 30;12(6):e00166-24. doi: 10.1128/spectrum.00166-24 (PMC11237744; doi:10.1128/spectrum.00166-24)
Supplement: Supplementary file 2 [file spectrum.00166-24-s0002.pdf]

Table S6. Strains and plasmids used in this study.

Strain/Plasmid	Genotype/Description	Reference/Source
Escherichia coli		
S17-1 (λpir)	recA, thi, pro, hsdR- M+RP4::2-Tc::Mu::Km Tn7 Tp^R Sm^R λpir	(1)
SM10 (λpir)	thi, thr, leu, tonA, lacY, supE, recA::RP4-2-Tc^R::Mu Km λpir	(2)
***Pseudomonas fluorescens* Pf0-1**		
SMC 4798	LapA-HA; HA tag inserted after residue 4093	(3)
SMC 8581	MapA-HA; HA tag inserted after residue 2601	(4)
SMC 9499	Δ*fleQ* LapA-HA	(5)
SMC 8746	Δ*fleQ* MapA-HA	(5)
SMC 9502	Δ*rsmA* LapA-HA	(5)
SMC 9503	Δ*rsmA*Δ*fleQ* LapA-HA	(5)
SMC 9507	Δ*rsmE* LapA-HA	(5)
SMC 9508	Δ*rsmE*Δ*fleQ* LapA-HA	(5)
SMC 9610	Δ*rsmI* LapA-HA	(5)
SMC 9611	Δ*rsmI*Δ*fleQ* LapA-HA	(5)
SMC 9858	Δ*rsmA*Δ*rsmE* LapA-HA	This Study
SMC 9859	Δ*rsmA*Δ*rsmE*Δ*fleQ* LapA-HA	This Study
SMC 9614	Δ*rsmA*Δ*rsmI* LapA-HA	(5)
SMC 9615	Δ*rsmA*Δ*rsmI*Δ*fleQ* LapA-HA	(5)
SMC 9860	Δ*rsmE*Δ*rsmI* LapA-HA	This study

SMC 9861	Δ*rsmE*Δ*rsmI*Δ*fleQ* LapA-HA	This study
SMC 9612	Δ*rsmA*Δ*rsmE*Δ*rsmI* LapA-HA	(5)
SMC 9613	Δ*rsmA*Δ*rsmE*Δ*rsmI*Δ*fleQ* LapA-HA	(5)
SMC 9862	Δ*rsmA*Δ*rsmE*Δ*rsmI*Δ*fliA* LapA-HA	This study
SMC 9863	Δ*rsmA*Δ*rsmE*Δ*rsmI*Δ*fleQ*Δ*fliA* LapA-HA	This study
SMC 9864	Δ*rsmA*Δ*rsmE*Δ*rsmI*Δ*gamA* LapA-HA	This study
SMC 9865	Δ*rsmA*Δ*rsmE*Δ*rsmI*Δ*fleQ*Δ*gamA* LapA-HA	This study
SMC 9866	Δ*rsmA*Δ*rsmE*Δ*rsmI*Δ*gamB* LapA-HA	This study
SMC 9867	Δ*rsmA*Δ*rsmE*Δ*rsmI*Δ*fleQ*Δ*gamB* LapA-HA	This study
SMC 9868	Δ*rsmA*Δ*rsmE*Δ*rsmI*Δ*gamC* LapA-HA	This study
SMC 9869	Δ*rsmA*Δ*rsmE*Δ*rsmI*Δ*fleQ*Δ*gamC* LapA-HA	This study
SMC 9870	Δ*rsmA*Δ*rsmE*Δ*rsmI*Δ*pleA* LapA-HA	This study
SMC 9871	Δ*rsmA*Δ*rsmE*Δ*rsmI*Δ*fleQ*Δ*pleA* LapA-HA	This study
SMC 9872	Δ*rsmA*Δ*rsmE*Δ*rsmI*Δ*pleB* LapA-HA	This study
SMC 9873	Δ*rsmA*Δ*rsmE*Δ*rsmI*Δ*fleQ*Δ*pleB* LapA-HA	This study
SMC 9874	Δ*rsmA*Δ*rsmE*Δ*rsmI*Δ*pleC* LapA-HA	This study
SMC 9875	Δ*rsmA*Δ*rsmE*Δ*rsmI*Δ*fleQ*Δ*pleC* LapA-HA	This study
SMC 9876	Δ*rsmA*Δ*rsmE*Δ*rsmI*; pMQ72-*rsmA*	This study
SMC 9877	Δ*rsmA*Δ*rsmE*Δ*rsmI*Δ*fleQ* LapA-HA; pMQ72-*rsmA*	This study
SMC 9702	Δ*rsmA*Δ*rsmE*Δ*rsmI* LapA-HA; pMQ72-*rsmE*	(5)
SMC 9703	Δ*rsmA*Δ*rsmE*Δ*rsmI*Δ*fleQ* LapA-HA; pMQ72-*rsmE*	(5)

**Plasmids**		
SMC 1210	pBT20	(6)
SMC 560	pEX18Tc	(7)
SMC 2765	pMQ30	(8)
SMC 2795	pMQ72	(8)
SMC 220	pSMC21	(9)
SMC 9850	pEX18Tc Δ*gamA*	This study
SMC 9851	pEX18Tc Δ*gamB*	This study
SMC 9852	pEX18Tc Δ*gamC*	This study
SMC 9854	pEX18Tc Δ*pleB*	This study
SMC 9855	pEX18Tc Δ*pleC*	This study
SMC 7975	pMQ30 Δ*fleQ*	(5)
SMC 9849	pMQ30 Δ*fliA*	This study
SMC 9853	pMQ30 Δ*pleA*	This study
SMC 9492	pMQ30 Δ*rsmA*	(10)
SMC 9506	pMQ30 Δ*rsmE*	(5)
SMC 9642	pMQ30 Δ*rsmI*	(5)
SMC 9856	pMQ72-*rsmA*	This study
SMC 9699	pMQ72-*rsmE*	(5)

Table S7. Primers used in this study.

Primer Name	Primer Sequence (5'-3')	Purpose
del fliA F1	gtcgactctagaggatccccgtgagcatggacagtttccgtatcg	Amplify region upstream of *fliA*
del fliA R1	cccacactgccttcatttcatagcacaggtcctgccg	Amplify region upstream of *fliA*
del fliA F2	aggacctgtgctatgaaatgaaggcagtgtggggacac	Amplify region downstream of *fliA*
del fliA R2	cgaattcgagctcggtacccgcgaaaaattgcgttgagcagatctg	Amplify region downstream of *fliA*
fliA seq F	caatctgttcgccaagttgaccaag	Verify Deletion of *fliA*
fliA seq R	gttggtgaacccgagatcacg	Verify Deletion of *fliA*
del gamA F1	gtcgactctagaggatccccgtgcgcgtggcgctcgat	Amplify region upstream of *gamA*
del gamA R1	gtcggctcagagcaagatccatctcacgtgatttggcgc	Amplify region upstream of *gamA*
del gamA F2	aatcacgtgagatggatcttgctctgagccgacaccgg	Amplify region downstream of *gamA*
del gamA R2	cgaattcgagctcggtaccccatcagttgcgcgtcttcgaagtc	Amplify region downstream of *gamA*
gamA seq F	tcgtgactgtacggtctagtg	Verify Deletion of *gamA*
gamA seq R	gggtggaaataacagccatcag	Verify Deletion of *gamA*
del gamB F1	gtcgactctagaggatcccctgctggacgcccacgatc	Amplify region upstream of *gamB*
del gamB R1	cagttcgatcacgttcacgggggtaacctgctgtaaattcgg	Amplify region upstream of *gamB*
del gamB F2	cagcaggttacccccgtgaacgtgatcgaactgttggc	Amplify region downstream of *gamB*

del gamB R2	cgaattcgagctcggtacccggccaggtggaacagactgg	Amplify region downstream of *gamB*
gamB seq F	gaccaggcaaaacagctggaag	Verify Deletion of *gamB*
gamB seq R	gtcacctgatcaagttcggcc	Verify Deletion of *gamB*
del gamC F1	gtcgactctagaggatcccccctcgctcatggtgacacc	Amplify region upstream of *gamC*
del gamC R1	gataaaaacttcaatcaggtcacaggacgatctccattctttcc	Amplify region upstream of *gamC*
del gamC F2	gagatcgtcctgtgacctgattgaagtttttatcctggaaccc	Amplify region downstream of *gamC*
del gamC R2	cgaattcgagctcggtaccccggaatcgtcagcaccgatttg	Amplify region downstream of *gamC*
gamC seq F	gatcagagtgctgccatcacc	Verify Deletion of *gamC*
gamC seq R	ccagccactgacccttcttg	Verify Deletion of *gamC*
del pleA F1	gtcgactctagaggatccccctacgaagtcgcgatgcaattgctc	Amplify region upstream of *pleA*
del pleA R1	aggttcgctcatgcaaatgaccacaattccgttcagggttg	Amplify region upstream of *pleA*
del pleA F2	cggaattgtggtcatttgcatgagcgaacctctgctg	Amplify region downstream of *pleA*
del pleA R2	cgaattcgagctcggtaccccagcatcagtttgttgacgttgtcttc	Amplify region downstream of *pleA*
pleA seq F	cgacgctggacgcatcg	Verify Deletion of *pleA*
pleA seq R	gcgctgaaagataaagccgaaatagtc	Verify Deletion of *pleA*
del pleB F1	gtcgactctagaggatccccgacgagagcacgcaaatgcg	Amplify region upstream of *pleB*
del pleB R1	cgccggtcagtcgctcatgcccctgccactttc	Amplify region upstream of *pleB*
del pleB F2	caggggcatgagcgactgaccggcgggcatg	Amplify region downstream of *pleB*

del pleB R2	cgaattcgagctcggtaccccaaaatcacgccatgagaccggttaatatg	Amplify region downstream of *pleB*
pleB seq F	caaatcggtgctgacgattccg	Verify Deletion of *pleB*
pleB seq R	gaaccgtgagatggaagtgtgc	Verify Deletion of *pleB*
del pleC F1	gtcgactctagaggatcccccagttcgacatagccaccgatg	Amplify region upstream of *pleC*
del pleC R1	aaggtgtaagaaaaaatgaccaaacagcctcgttcacgaatgag	Amplify region upstream of *pleC*
del pleC F2	gaacgaggctgtttggtcattttttcttacaccttttgtcgccag	Amplify region downstream of *pleC*
del pleC R2	cgaattcgagctcggtacccggcactaattcgggagggtatctg	Amplify region downstream of *pleC*
pleC seq F	catcaccttgtcagtgcgtgag	Verify Deletion of *pleC*
pleC seq R	cttgccgtacttgtacgacagttc	Verify Deletion of *pleC*
pMQ72 *rsmA* F	cgaattcgagctcggtacccgaaggagatatacatatgctgattctgactcgtcggtg	Amplify *rsmA*
pMQ72 *rsmA R*	gtcgactctagaggatccccttaatggcttggttcttcgtccttc	Amplify *rsmA*
pMQ72 *rsmE* F	cgaattcgagctcggtacccgaaggagatatacatatgctgatactcacccgcaaagtc	Amplify *rsmE*
pMQ72 *rsmE* R	gtcgactctagaggatcccctcagggcgtttgtggcttgtc	Amplify *rsmE*
pMQ30 seq F	gatgcctggcagttccctactctcg	Sequencing pMQ30/pEX18Tc MCS
pMQ30 seq R	ctggcacgacaggtttcccgactg	Sequencing pMQ30/pEX18Tc MCS
pMQ72 seq F	gaattgtgagcggataacaatttcacacag	Sequencing pMQ72 MCS
pMQ72 seq R	gacaactccagtgaaaagttcttctcctttac	Sequencing pMQ72 MCS
Rnd1_pBT20Tn	tataatgtgtggaattgtgagcgg	Arbitrary PCR
Rnd2_pBT20Tn	acaggaaacaggactctagagg	Arbitrary PCR
Seq_pBT20Tn	cacccagctttcttgtacac	Arbitrary PCR
GAO_ARB1	ggccacgcgtcgactagtacnnnnnnnnnngatat	Arbitrary PCR

GAO_ARB2	ggccacgcgtcgactagtac	Arbitrary PCR

Literature Cited

1. de Lorenzo V, Cases I, Herrero M, Timmis KN. 1993. Early and late responses of TOL promoters to pathway inducers: identification of postexponential promoters in *Pseudomonas putida* with *lacZ-tet* bicistronic reporters. J Bacteriol 175(21):6902–6907.

2. Miller VL, Mekalanos JJ. 1988. A novel suicide vector and its use in construction of insertion mutations: osmoregulation of outer membrane proteins and virulence determinants in *Vibrio cholerae* requires *toxR*. J Bacteriol 170(6):2575–2583.

3. Monds RD, Newell PD, Gross RH, O'Toole GA. 2007. Phosphate-dependent modulation of c-di-GMP levels regulates *Pseudomonas fluorescens* Pf0-1 biofilm formation by controlling secretion of the adhesin LapA. Mol Microbiol 63(3):656–679.

4. Collins AJ, Pastora AB, Smith TJ, O'Toole GA. 2020. MapA, a second large RTX Adhesin conserved across the pseudomonads, contributes to biofilm formation by *Pseudomonas fluorescens.* J Bacteriol 202(18):e00277-20.

5. Pastora AB, O'Toole GA. 2023. The regulator FleQ both transcriptionally and post-transcriptionally regulates the level of RTX adhesins of *Pseudomonas fluorescens*. J Bacteriol 205(9):e00152-23.

6. Kulasekara HD, Ventre I, Kulasekara BR, Lazdunski A, Filloux A, Lory S. 2005. A novel two-component system controls the expression of *Pseudomonas aeruginosa* fimbrial cup genes. Mol Microbiol 55(2):368–380.

7. Hoang TT, Karkhoff-Schweizer RR, Kutchma AJ, Schweizer HP. 1998. A broad-host-range Flp-FRT recombination system for site-specific excision of chromosomally-located DNA

sequences: application for isolation of unmarked *Pseudomonas aeruginosa* mutants. Gene 212(1):77–86.

8. Shanks RMQ, Caiazza NC, Hinsa SM, Toutain CM, O'Toole GA. 2006. *Saccharomyces cerevisiae*-based molecular tool kit for manipulation of genes from gram-negative bacteria. Appl Environ Microbiol 72(7):5027–5036.

9. Cowan SE, Gilbert E, Khlebnikov A, Keasling JD. 2000. Dual labeling with green fluorescent proteins for confocal microscopy. Appl Environ Microbiol 66(1):413–418.

10. Evans AF, Wells MK, Denk J, Mazza W, Santos R, Delprince A, Kim W. 2022. Spatial structure formation by RsmE-regulated extracellular secretions in *Pseudomonas fluorescens* Pf0-1. J Bacteriol 204(10):e00285-22.
